# Allele-specific methylation, and InDels of *PmMYB10.5b* induced by alternative splicing, participate in regulating the leaf color change in *Prunus mume* ‘Meiren’

**DOI:** 10.1093/hr/uhag039

**Published:** 2026-02-18

**Authors:** Juan Meng, Ziwei Li, Haoning Wang, Zhiyi Yue, Zimo Li, Guijia Wang, Tangren Cheng, Qixiang Zhang, Lidan Sun

**Affiliations:** Beijing Key Laboratory of Ornamental Plants Germplasm Innovation and Molecular Breeding, State Key Laboratory of Efficient Production of Forest Resources, National Engineering Research Center for Floriculture, Beijing Laboratory of Urban and Rural Ecological Environment, School of Landscape Architecture, Beijing Forestry University, Beijing 100083, China; Beijing Key Laboratory of Ornamental Plants Germplasm Innovation and Molecular Breeding, State Key Laboratory of Efficient Production of Forest Resources, National Engineering Research Center for Floriculture, Beijing Laboratory of Urban and Rural Ecological Environment, School of Landscape Architecture, Beijing Forestry University, Beijing 100083, China; Beijing Key Laboratory of Ornamental Plants Germplasm Innovation and Molecular Breeding, State Key Laboratory of Efficient Production of Forest Resources, National Engineering Research Center for Floriculture, Beijing Laboratory of Urban and Rural Ecological Environment, School of Landscape Architecture, Beijing Forestry University, Beijing 100083, China; Beijing Key Laboratory of Ornamental Plants Germplasm Innovation and Molecular Breeding, State Key Laboratory of Efficient Production of Forest Resources, National Engineering Research Center for Floriculture, Beijing Laboratory of Urban and Rural Ecological Environment, School of Landscape Architecture, Beijing Forestry University, Beijing 100083, China; Beijing Key Laboratory of Ornamental Plants Germplasm Innovation and Molecular Breeding, State Key Laboratory of Efficient Production of Forest Resources, National Engineering Research Center for Floriculture, Beijing Laboratory of Urban and Rural Ecological Environment, School of Landscape Architecture, Beijing Forestry University, Beijing 100083, China; Beijing Key Laboratory of Ornamental Plants Germplasm Innovation and Molecular Breeding, State Key Laboratory of Efficient Production of Forest Resources, National Engineering Research Center for Floriculture, Beijing Laboratory of Urban and Rural Ecological Environment, School of Landscape Architecture, Beijing Forestry University, Beijing 100083, China; Beijing Key Laboratory of Ornamental Plants Germplasm Innovation and Molecular Breeding, State Key Laboratory of Efficient Production of Forest Resources, National Engineering Research Center for Floriculture, Beijing Laboratory of Urban and Rural Ecological Environment, School of Landscape Architecture, Beijing Forestry University, Beijing 100083, China; Beijing Key Laboratory of Ornamental Plants Germplasm Innovation and Molecular Breeding, State Key Laboratory of Efficient Production of Forest Resources, National Engineering Research Center for Floriculture, Beijing Laboratory of Urban and Rural Ecological Environment, School of Landscape Architecture, Beijing Forestry University, Beijing 100083, China; Beijing Key Laboratory of Ornamental Plants Germplasm Innovation and Molecular Breeding, State Key Laboratory of Efficient Production of Forest Resources, National Engineering Research Center for Floriculture, Beijing Laboratory of Urban and Rural Ecological Environment, School of Landscape Architecture, Beijing Forestry University, Beijing 100083, China

## Abstract

*Prunus mume* ‘Meiren’, a member of the Meiren cultivar group, is a valuable ornamental woody plant prized for its purple flowers and leaves. However, its leaf color exhibits instability during the growth and development and the underlying mechanisms remain unclear. In our study, we conducted genome-wide methylation analysis on leaves at different developmental stages to investigate the role of methylation patterns and allele-specific methylation (ASM) in leaf color change. Results revealed a significant increase in CHH methylation during leaf development, suggesting its responsiveness to environmental factors and dynamic association with color changes. Notably, CG methylation was imbalanced between the ‘Meiren’ haplotype M (HM) and haplotype C (HC), with the HM subgenome showing higher methylation levels, particularly in promoter regions of key anthocyanin-related genes like *PmMYB10.5*, where ASM negatively correlated with allele-specific expression. Additionally, we identified two alternative splicing variants of *PmMYB10.5b,* named *PmMYB10.5b1* (*PmMYB10.5b*  ^*△I24*^) and *PmMYB10.5bP* (*PmMYB10.5b*  ^*△D10*^), respectively. Both the InDel mutations altered the R2 domain structure of the MYB protein. Functional assays demonstrated that these variants lost transcriptional activation ability and failed to promote anthocyanin biosynthesis. Instead, they may compete with the PmMYB10.5b for binding to the PmbHLH3, disrupting regulatory complexes in the anthocyanin pathway and exerting inhibitory effects. These results augment our understanding of the epigenetic and genetic factors influencing leaf color change in ‘Meiren’ and provide novel insights into its regulatory mechanisms.

## Introduction

Interspecific hybridization is a common breeding strategy that integrates allelic gene pools and facilitates evolutionary progress [1, 2]. Heterosis, also known as hybrid vigor, refers to a phenomenon in which hybrid offspring display enhanced performance in certain traits compared to one or both parents [3]. This superiority is largely attributed to the complementary interactions between the parental genomes [4]. Studies have revealed that differential expression regulation of alleles often leads to dominant or overdominant effects in specific traits of the offspring, which serves as one of the key theoretical explanations for heterosis [5]. Notably, expression differences and methylation differences of alleles inherited from the parental lines play crucial roles in shaping phenotypic outcomes [6]. Allele-specific expression (ASE), in which alleles inherited from parental genomes are differentially expressed in specific tissues or developmental stages of hybrids, serves as a key mechanism underlying phenotypic variation and heterosis [5]. This phenomenon has been extensively studied in model crops such as rice, where ASE genes (ASEGs) are primarily classified into two categories: consistent ASEGs that exhibit persistent expression bias toward one subgenome across various tissues or developmental stages, and inconsistent ASEGs, in which alleles show context-dependent expression bias, where preferential expression from one subgenome occurs only in specific spatial or temporal conditions [4].

DNA methylation is an essential epigenetic modification that orchestrates critical processes including gene regulation, development, and adaptation to the environments [7]. In plants, DNA methylation is crucial for various biological processes, like genomic imprinting, silencing of gene expression, and responses to environmental stress [8–10]. Emerging evidence underscores the involvement of DNA methylation in regulating the biosynthesis and metabolism of secondary metabolites, such as anthocyanins—key compounds influencing plant pigmentation and stress adaptation [11–14]. The biosynthesis and metabolism of anthocyanins in plants are directly catalyzed by enzyme genes such as *CHI*, *CHS*, *F3H*, *DFR* [15, 16], and regulated by a variety of transcription factors, such as MBW (MYB-bHLH-WD40) [17, 18]. There have been many reports on the involvement of DNA methylation modification in the regulation of color of plant organs. In the chrysanthemum, *CmMYB6* is an epiallele associated with anthocyanin biosynthesis. CHH-type methylation modification in the promoter region of the *CmMYB6* gene leads to flower color changes, which is stable in the offspring of chrysanthemum and can be genetically involved in determining the flower color of chrysanthemum [19, 20]. In the formation of petal blotch in tree peony, hypermethylation of promoters represses the expression of anthocyanin biosynthesis genes (ABGs), likely *PrANS*, in nonblotch areas. The methylation dynamics of ABGs promoter during flower development contributed to their particular expression solely in the petal [21]. Additionally, DNA methylation plays a regulatory role in fruit peel pigmentation, as evidenced in horticultural crops including apples and pear [22, 23]. In ‘Daihong’ apple cultivar, CHH-type methylation was increased during fruit development. Hypermethylation in promoter regions of flavonoid biosynthesis genes is associated with the reduction of gene expression and flavonoid content, reducing the fruit skin color fading during the apple development [22]. For pear skin color, the hypomethylated CHH context was detected in the anthocyanin biosynthesis genes, likely *PyUFGT*, *PyPAL*, *PyDFR*, and *PyANS*, which enhanced transcriptional activation and promoted anthocyanin accumulation after light re-exposure [23].

Alternative splicing is a regulatory mechanism whereby exons from a single precursor mRNA are rearranged and joined in different combinations to generate two or more distinct mature mRNA variants. This process serves as a crucial strategy for expanding proteomic diversity and enables precise post-transcriptional control of gene expression [24, 25]. Alternative splicing is a fundamental biological process that generates transcriptomic and proteomic diversity. The major splicing types include: constitutive splicing, exon skipping, alternative 3′ splice site, alternative 5′ splice site, intron retention, and mutually exclusive exons [25]. In plants, this process plays pivotal roles in regulating various developmental and growth stages including embryogenesis, flowering time, fruit ripening, and leaf senescence, while also serving critical functions in responding to abiotic stresses (drought, extreme temperatures, salinity) and biotic stresses [26]. Research demonstrates that alternative splicing acts as a molecular sensor for environmental changes, such as heat shock (HS), [27] and OsbZIP58 [28] for heat stress, AtSRAS1 for salt stress [8], and ZmCCA1 for drought stress [29]. These alternative splicing variants enhance the diversity of gene expression and protein function, probably conferring phenotypic plasticity in response to environments, which represents a crucial regulatory mechanism enabling plants to adapt to complex and changing environments.

*Prunus mume*, one of China's celebrated traditional flowers, has been cultivated for over 3000 years. As an important early-spring woody ornamental species, it holds significant aesthetic, economic, and cultural value. *P. mume* ‘Meiren’, belonging to the Meiren Group [30], is an interspecific hybrid developed from a cross between *P. cerasifera* ‘Pissardii’ and *P. mume* Pink Double Group. This hybrid combines the superior traits of both parents, exhibiting purple pigmentation in stems, leaves, flowers, and fruits, making it a unique ornamental tree valued for both foliage and floral display [31]. In northern China, where colorful leaf plants are essential for enriching landscape diversity, breeding *P. mume* cultivars with vibrant leaf coloration has become a key horticultural objective. Overall, the success of ‘Meiren’ demonstrates the potential of interspecific hybridization in developing novel ornamental varieties.

In our previous study, we performed haplotype-resolved genome sequencing of *P. mume* ‘Meiren’ and identified a key inversion (INV) variant and critical transcription factor PmMYB10.5b associated with its purple color trait on the haplotype M (HM) [31]. However, we observed that the purple leaves of ‘Meiren’ exhibit a regreening phenomenon during the development, suggesting that leaf color potentially associated with seasonal environmental changes. We hypothesize that the external factors during the leaf development may modulate this trait through DNA methylation changes, which could directly or indirectly participate in phenotypic regulation. In this study, we conducted whole-genome bisulfite sequencing (WGBS) on leaves at five different developmental stages to investigate allele-specific methylation (ASM) patterns and their influence on purple leaf coloration in *P. mume* ‘Meiren’. Our results revealed that differential DNA methylation levels in the *PmMYB10.5* alleles, particularly within promoter regions, along with alternatively spliced transcript variants generated from the *PmMYB10.5b* coding sequence, collectively contribute to functional diversification of this transcription factor and serve as one of the primary mechanisms underlying both the functional diversification and the observed leaf regreening phenomenon. These epigenetic and post-transcriptional regulatory layers work synergistically to modulate the purple leaf pigmentation in response to developmental and environmental cues. By elucidating these epigenetic mechanisms, we hope to provide new insights into the molecular basis of purple leaf color variation.

## Results

### The continuous increase in CHH methylation levels during the developmental stage of ‘Meiren’ leaf

**Figure 1:**
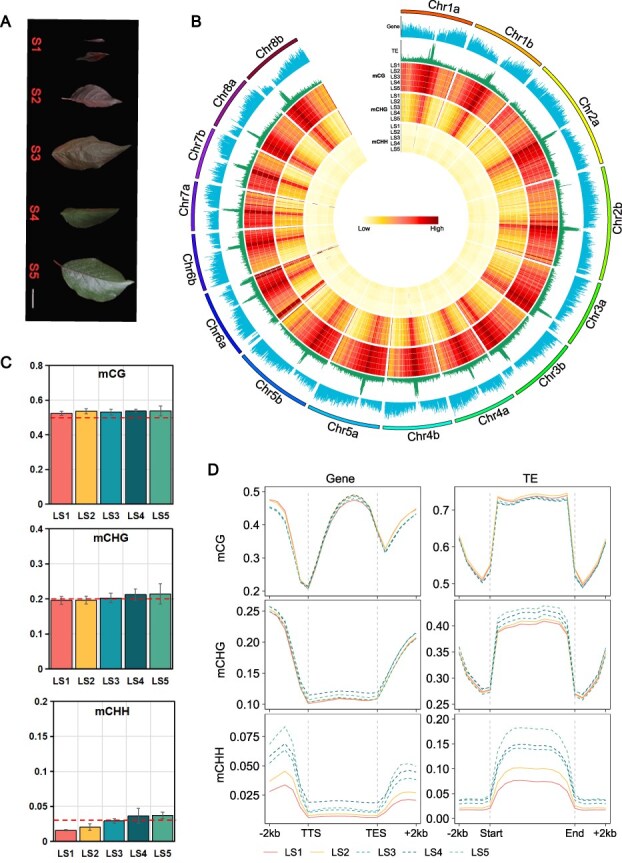
DNA methylation levels at five leaf developmental stages of ‘Meiren’. (A) Leaf tissues at five developmental stages (S1-S5). (B) Genome wide methylation features of ‘Meiren’. (C) CG, CHG, and CHH DNA methylation levels at five leaf developmental stages of ‘Meiren’. (D) Distribution of CG, CHG and CHH DNA methylation at gene body and TE in leaf developmental stages of ‘Meiren’.

‘Meiren’ is obtained through interspecific hybridization between the purple-leaved *P. cerasifera* ‘Pissardii’ and *P. mume* Pink Double Group [31]. It combines the excellent traits of both parents, exhibiting a purple appearance in leaves, flowers, and fruit. However, unlike its maternal parent, the purple leaf color of ‘Meiren’ shows instability during development, which may be influenced by environmental factors (Fig. 1A). DNA methylation modifications are closely related to these environmental factors. In order to investigate the effects of DNA methylation on leaf color and leaf development, we performed genome-wide methylation analysis at the five developmental stages of ‘Meiren’ leaves (Fig. 1A). The DNA methylation characteristics of the whole genome of ‘Meiren’ indicate that there are significant differences in methylation sites throughout the genome (Fig. 1B).

The overall DNA methylation levels were increased during developmental stages, particularly with a significant increase at CHG and CHH sites in the LS5 (Fig. 1C, Table S1). We therefore separately analyzed the levels of CG, CHG, and CHH methylation in gene bodies, promoter regions, and transposable element (TE) regions (Fig. S1, Table S2). The results demonstrated a progressive increase in CHG and CHH methylation levels across gene bodies, promoters, and TE regions during leaf development, with the most significant upregulation occurring in CHH context. Additionally, we focused on the methylation levels in the gene body and TE, as well as in the 2-kb upstream and downstream regions (Fig. 1D). Methylation levels at CG sites did not exhibit significant changes. In contrast, CHG methylation differences were primarily observed in gene bodies and TE regions. At CHH sites, methylation levels displayed significant variations upstream of the transcription termination site (TTS) and downstream of gene bodies, progressively increasing as leaves transitioned into maturity and the color-changing phase, peaking at the fifth developmental stage. Notably, methylation changes in TE bodies were markedly more pronounced than those in upstream and downstream regions. Thus, CHH methylation may be highly responsive to environmental factors during leaf development, exhibiting dynamic changes correlated with leaf color transition.

In summary, methylation levels in ‘Meiren’ leaves undergo substantial alterations from the early tender stage to full maturity, with CHH methylation displaying the most pronounced increase. This pattern suggests a potential link between CHH methylation dynamics and external environmental fluctuations.

### Differential DNA methylation at CHH affects gene expression in ‘Meiren’

**Figure 2:**
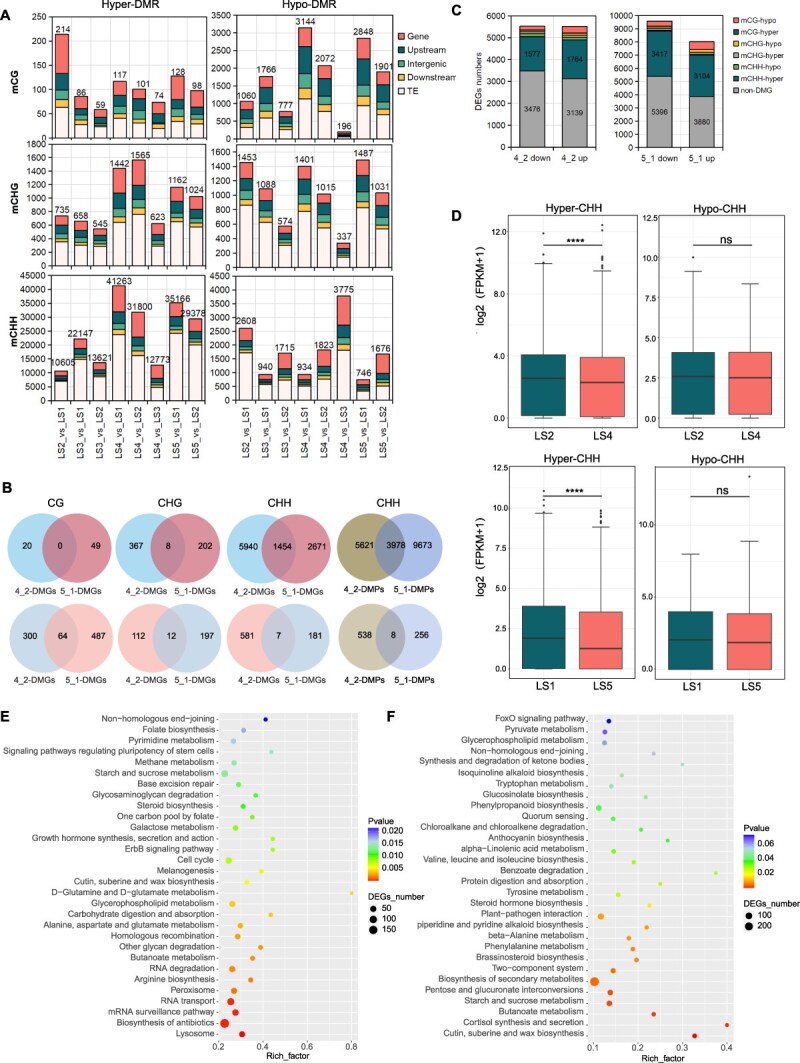
Analysis of DNA methylation differences in different leaf developmental stages. (A) Numbers of DMRs of 200 bp sliding-window with 50 bp as step-size between stages. DMRs are categorized by genomic context: gene body, upstream 2 kb, downstream 2 kb, TE, and intergenic regions. (B) Venn diagrams showing overlapping DMGs and differentially methylated promoters (DMPs) in CG, CHG, and CHH contexts between LS5 vs. LS1 and LS4 vs. LS2. (C) Distribution of CG, CHG and CHH hyper- and hypo-DMRs in DEGs at LS5 vs. LS1 and LS4 vs. LS2. (D) Expression levels (log2(FPKM +1)) of hyper- or hypo-CHH DMGs at LS5 vs. LS1 and LS4 vs. LS2. Data are shown as mean ± SD from three biological replicates. Statistical significance was determined using a one-tailed t-test (^****^P < 0.00001; ns, not significant). The KEGG enrichment of CHH DMR in LS4 vs. LS2 (E) and LS5 vs. LS1(F).

To investigate the role of differentially methylated regions (DMRs) in leaf development of *P. mume* ‘Meiren’, we performed comprehensive DMR analysis across all three sequence contexts (CG, CHG, and CHH), examining gene bodies, 2-kb upstream and downstream flanking regions, intergenic regions, and TEs (Fig. S2). The results indicated that the majority of DMRs are distributed in the TE regions, followed by the gene bodies and upstream regions, less in downstream and intergenic regions. In the CG context, the number of hyper-DMRs is significantly greater than hypo-DMRs when comparing different stages, especially in the later developmental stages (LS4 and LS5) compared to LS1. However, the number of hyper-DMRs significantly increased in the CHG context, with a smaller difference compared to the number of hypo-DMRs. Most strikingly, the number of DMRs in the CHH context increases dramatically, particularly for hyper-DMRs. At least 30 000 CHH hyper-DMRs were detected in the LS4 and LS5 stages compared to the LS1 and LS2 stages, representing that hyper-CHH methylation differences are the primary type of methylation changes during the development of ‘Meiren’ leaves (Fig. 2A, Table S3). Notably, LS4 exhibited the highest number of hyper-DMRs among all developmental stages (Fig. 2A). Therefore, we focused on analyzing methylation differential regions by selecting the initial developmental stage apical leaf (LS1) and the mature apical leaf (LS5), as well as the apical leaf (LS2) and the lower mature leaf (LS4) from the same branch. About 35% of CHH hypermethylation differentially methylated genes (DMGs) in LS5 vs. LS1 was overlap with those in LS4 vs. LS2, while there was almost no overlap in the number of hypo-DMGs. Similarly, 41% of CHH hyper-DMRs in gene promoters in LS4 vs. LS2 overlap with those in LS5 vs. LS1. The number of overlapping DMRs for CG and CHG methylation types is relatively small in both comparisons (Fig. 2B). This demonstrates that, regardless of whether they are apical or differently positioned leaves, CHH-type hypermethylation differences may play an important role in the development of ‘Meiren’ leaves.

To study the impact of DNA methylation on gene expression, we also performed transcriptome sequencing on leaves from the same period in our previous research [31]. PCA indicated that gene expression abundance was significantly differentiated at each stage (Fig. S3). Further analysis of methylation patterns in differentially expressed genes (DEGs) from the LS5 vs. LS1 and LS4 vs. LS2 comparsions revealed that 40% to 50% showed methylation differences in either gene bodies or 2-kb promoter regions during leaf color transition (Fig. 2C). To further investigate the relationship between DMGs and DEGs, especially in CHH context, we analyzed the expression abundance of DMGs in LS5 vs. LS1 and LS4 vs. LS2 (Fig. 2D). Our analysis revealed that genes with hyper-CHH DMGs in the later developmental stages (LS4 and LS5) displayed significantly lower expression abundance compared to their counterparts in early stages (LS1 and LS2), demonstrating an inverse correlation between CHH methylation levels and gene expression (Fig. 2D). However, there was no significant relationship between CG and CHG methylation and gene expression (Fig. S4). Complementary KEGG pathway enrichment analysis of these DMGs showed significant representation in biological processes related to lysosome function, cell wall metabolism, and anthocyanin biosynthesis (Fig. 2E and F). These findings collectively suggest that dynamic CHH methylation differences during leaf development may serve as an important regulatory mechanism in the transcriptional repression of specific gene sets and changes of leaf pigmentation and developmental transition from young to mature leaves in ‘Meiren’.

### CG methylation imbalance and ASM are associated with ASE

**Figure 3:**
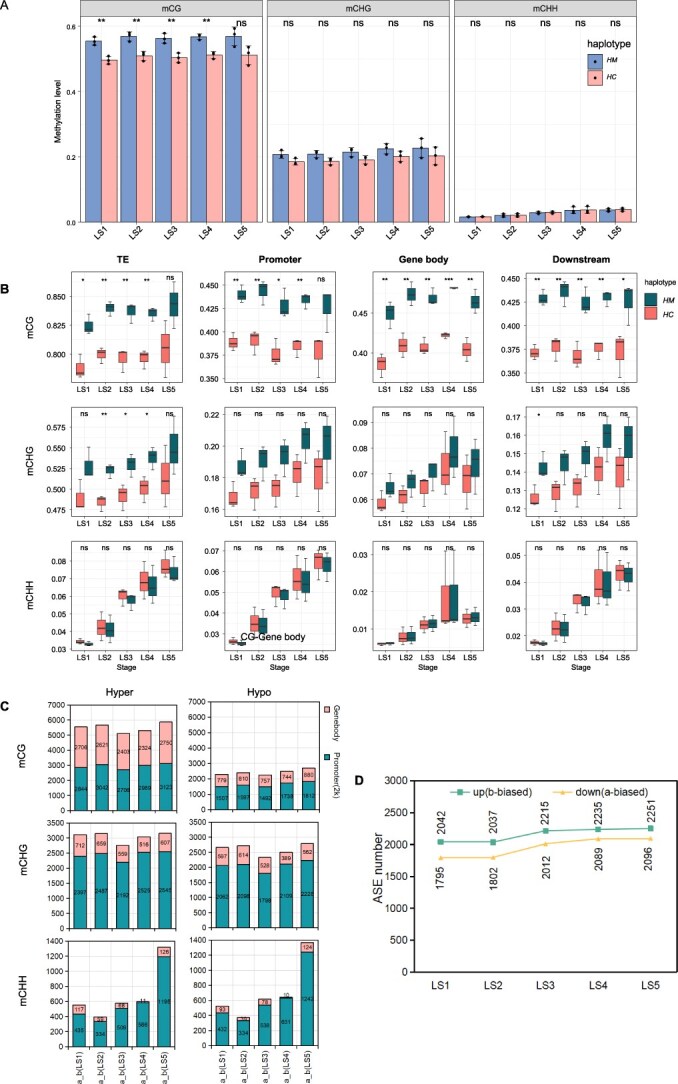
CG methylation imbalance between ‘Meiren’ HM and HC subgenomes. (A) Methylation level distribution of HM and HC subgenomes at CG, CHG, and CHH sites. (B) CG, CHG, and CHH methylation levels of TE, promoter, gene body, and downstream in HM and HC subgenomes at five leaf developmental stages. (C) Number of hyper-(a-biased) or hypo-(b-biased) allelic-specific methylation at CG, CHG, and CHH sites in five leaf stages. (D) Number of ASEG biased in haplotype M (a-biased) and biased in haplotype C (b-biased) at leaf developmental stages. Data in **A** and **B** are shown as mean ± SD of three biological replicates. One-tailed *t* test was used for statistical significance test (^*^P < 0.05, ^**^P < 0.01, ^***^P < 0.001, ns, not significant).

Subsequently, we focused on the methylation levels of subgenomes and the changes in methylations of alleles. The CG, CHG, and CHH methylation levels were statistically analyzed in the HC and HM subgenomes according to leaf developmental stages. Unlike the overall trend of methylation changes at the whole-genome level, there was a significant difference in CG methylation levels between the HM and HC subgenomes in ‘Meiren’. The methylation level of the HM subgenome was significantly higher than that of the HC subgenome throughout the leaf development process. CHG methylation levels showed a similar trend between two subgenomes, but the difference was not significant, while CHH methylation levels were almost identical between the two haplotypes (Fig. 3A). Further analysis of CG, CHG, and CHH methylation levels in gene regions, promoter regions, TE regions, and downstream regions was conducted (Fig. 3B). The results revealed that CG methylation levels were consistently higher in the HM subgenome than in the HC subgenome and tended to stabilize during leaf developmental stages. For CHG methylation, the HM subgenome exhibited higher methylation levels than the HC subgenome across all four sequence types, but the difference was not significant. In contrast, the continuously increasing CHH methylation levels during development showed no significant differences between the haplotypes.

We next examined DMRs between alleles by analyzing ASM patterns (Fig. 3C). In CG methylation, the number of ASM sites biased toward the HM allele (allele a-biased) was significantly higher than those biased toward the HC allele (allele b-biased) in both gene bodies and promoter regions. In contrast, for CHG and CHH methylation types, there was no significant difference in the number of ASM sites biased toward either haplotype. Comparing gene bodies and promoter regions, ASM sites of all three methylation types were more dominant in promoter regions, particularly for CHH methylation. These results indicate that CG-type ASM exhibits significant differences between two subgenomes of ‘Meiren’, with a specific bias toward the HM subgenome and a clear imbalance (Fig. 3C). In contrast, the number of ASE events was higher in the HC subgenome than in the HM subgenome, showing an opposite trend to that of ASM (Fig. 3D).

Additionally, functional enrichment analysis was performed on ASM regions at different developmental stages. In the LS5 stage, genes exhibiting CG-type ASM were primarily enriched in biosynthetic and metabolic pathways such as diarylheptanoid and gingerol biosynthesis, nitrogen metabolism, and flavonoid biosynthesis (Fig. S5).

**Figure 4:**
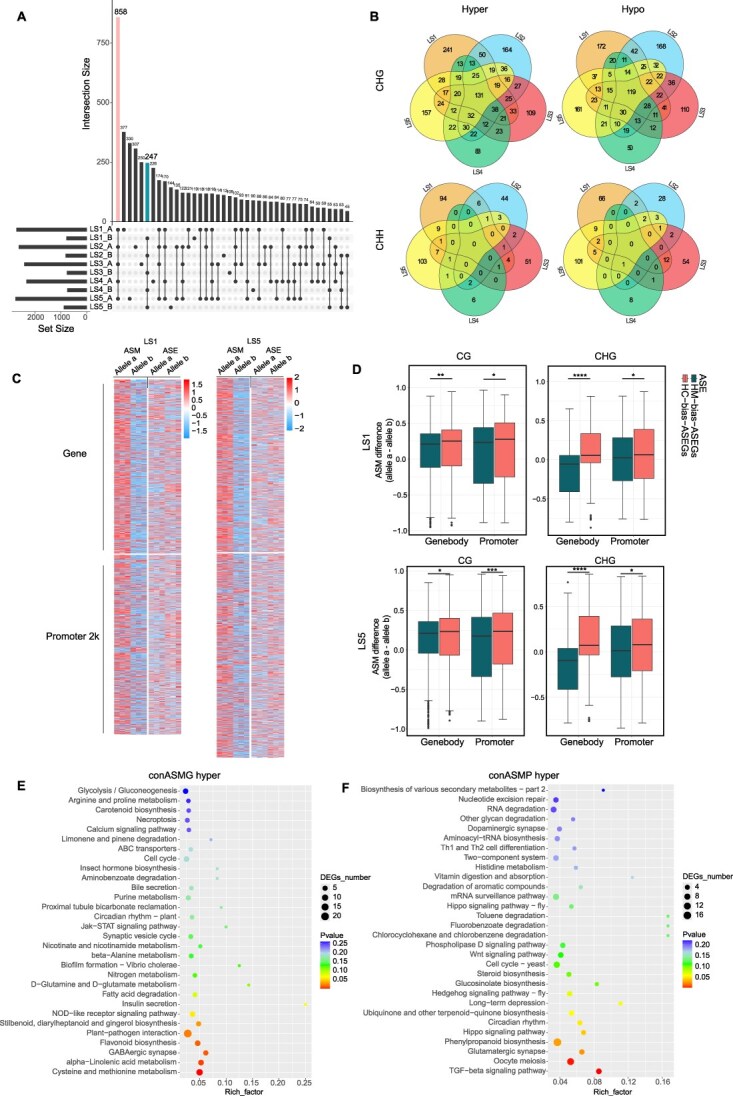
ASM are associated with ASE. (A) CG ASM numbers of gene body at five leaf developmental stages. The upset plot was drawn by UpSetR package. Only top 40 categories are shown panel A in Fig. 4A represents alleles from HM. B in Fig. 4A represents alleles from HC. (B) Venn diagrams showing overlapping CHG and CHH ASM numbers of gene body at five leaf developmental stages. (C) Allele-specific expression and ASM heat map of LS1 and LS5 in gene regions and 2-kb region of promoters. (D) ASM differences (allele a minus allele b) of a-biased ASEGs and b-biased ASEGs in gene body and promoter 2 kb of ‘Meiren’ leaf stages. Data are shown as mean ± SD of three biological replicates. Statistical significance was determined using a one-tailed *t*-test (^*^P < 0.05, ^**^P < 0.01, ^***^P < 0.0001, ^****^P < 0.00001). KEGG enrichment of consistent ASMG (E) and ASMP (F).

Previous studies have shown that allelic methylation differences can impact ASEs [32]. Therefore, we analyzed the distribution of ASM in gene bodies (ASMG) and promoters (ASMP) for CG, CHG, and CHH contexts across leaf developmental stages (Fig. 4A and B). We defined alleles showing consistent methylation bias (either toward the HM or HC subgenome) across all five developmental stages as exhibiting consistent ASM. In CHG methylation, the numbers of consistent ASMGs biased toward HM and HC were 131 and 119, respectively, showing no significant difference, and 782 (HM-biased) and 560 (HC-biased) for ASMPs. In CHH methylation, almost no alleles displayed consistent ASM (Fig. 4B). In CG methylation, there were 1,105 pairs consistent ASMGs, of which 858 (77.6%) were biased toward the HM subgenome (Fig. 4A). Similarly, among 1,647 pairs ASMPs, the majority were located in the HM subgenome (Figs S6 and S7). These results demonstrate a significant imbalance in ASM distribution between the HM and HC subgenomes.

The methylation difference of genes and their promoters directly influences transcriptional expression and gene function. To investigate the potential correlation between ASM and ASE, we performed association analyses based on allelic information and conducted quantitative assessments of methylation differences in ASEGs. Our results revealed an inverse relationship between methylation and expression patterns during leaf development. Specifically, in both early-stage purple leaves and mature faded-green leaves, a subset of genes with ASM showed opposite trends between their methylation status and expression levels (Fig. 4C). Further analysis of ASEGs demonstrated distinct methylation patterns depending on allelic expression bias: in HC-biased ASEGs (b-bias-ASEGs, Fig. 4D), the HM allele displayed significantly higher CG and CHG methylation levels in both gene bodies and promoter regions compared to the HC allele. Conversely, HM-biased ASEGs (a-bias-ASEGs, Fig. 4D) showed lower CG and CHG methylation levels. These findings clearly indicate a negative correlation between ASM at CG and CHG sites and ASE, which aligns with the well-established repressive role of DNA methylation in gene expression regulation. In conclusion, our study confirms that allele-specific CG and CHG methylation patterns show a negative correlation with allelic gene expression during leaf development in ‘Meiren’, suggesting these epigenetic modifications play an important regulatory role in leaf biological process.

Then, Venn diagram analysis of alleles exhibiting both ASM and ASE in LS1 and LS5 stages revealed that approximately 30% of genes with ASM in gene bodies and 35% of alleles with ASM in promoters showed ASE patterns. Functional enrichment analysis indicated that genes with CG-type consistent ASM in ASMGs and ASMPs were primarily associated with biosynthetic pathways, including cysteine and methionine metabolism, α-linolenic acid metabolism, phenylpropanoid biosynthesis, and flavonoid biosynthesis (Fig. 4E and F). In summary, we proposed that the methylation difference of ‘Meiren’ was one of the significant motives influencing allelic gene expressions and leaf development and metabolic processes such as flavonoid biosynthesis. Firstly, a genome-wide CG methylation imbalance between ‘Meiren’ HM and HC might be inheritable factors from its parents. However, CHH methylation primarily responded to environmental factors and showed dynamic differences along with the change of leaf color.

### DNA methylation is involved in regulating the metabolism of flavonoid in leaves

**Figure 5:**
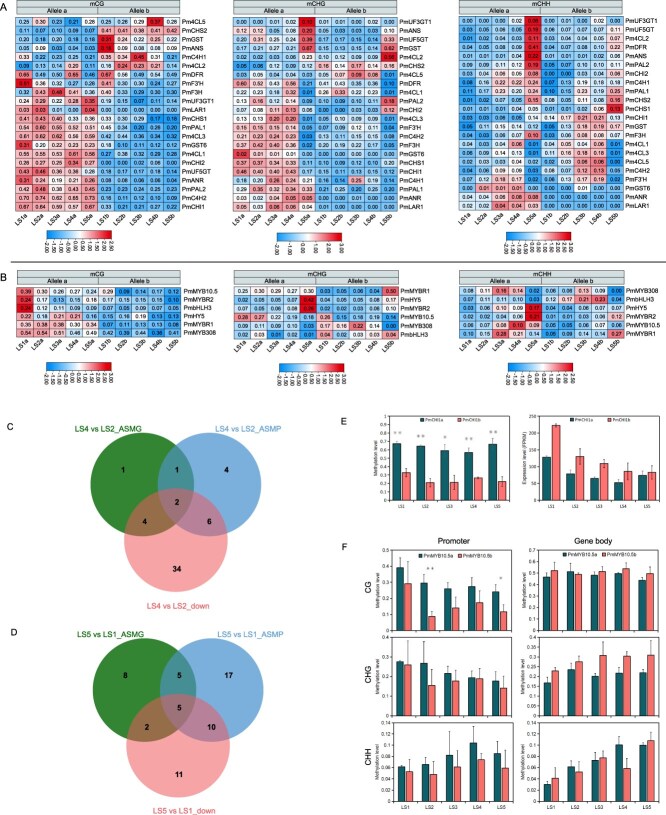
DNA methylation is involved in regulating the metabolism of flavonoid in leaves. Heatmap of methylation levels in the promoters of ABGs (A) and transcription factor (B) alleles. Venn diagram showing the overlap between differentially CHH methylated genes and differentially expressed genes in gene and promoter regions during leaf developmental stages LS4 vs. LS2 (C) and LS5 vs. LS1 (D). (E) CG methylation levels in the promoter region and gene expressions of *PmCHI1* alleles*.* (F) Methylation levels in the promoter region and gene body of *PmMYB10.5* alleles. Statistical significance was determined using a one-tailed *t*-test (^*^P < 0.05, ^**^P < 0.01).

During leaf development, KEGG functional enrichment analysis of DMRs and ASMs with color variations revealed significant enrichment in pathways related to flavonoid and anthocyanin biosynthesis (Figs 2E and 4E). Therefore, to clarify the influence of DNA methylation on leaf color variation, we analyzed the methylation levels of genes associated with flavonoids. In CG methylation, Approximately half of the ABG alleles derived from the HM subgenome exhibited higher methylation levels in gene body across most developmental stages compared to those from the HC subgenome, particularly in upstream genes of the biosynthesis pathway (*Pm4CL3*, *Pm4CL5*, *PmC4H1*, *PmC4H2*, *PmCHI1*) and downstream genes (*PmF3H*, *PmANS*) (Fig. S8A). For promoter regions, CG methylation levels were significantly elevated, and the differences between alleles became more pronounced. Most alleles derived from the HM subgenome showed higher methylation levels than those from the HC subgenome (e.g., *PmPAL1*, *PmPAL2*, *Pm4CL3*, *PmC4H2*, *PmCHS1*, *PmCHI1/2*, *PmUF3GT1*). This methylation trend was less evident in the CHG context, whereas in the CHH context, inter-allelic methylation differences gradually diminished. During leaf development, the CHH methylation levels of most ABGs continuously increased, peaking in the later stages (LS4 and LS5). These notable differences were observed in genes, such as *PmDFR*, *PmANS*, *Pm4CL3*, *PmPAL1/2*, *PmC4H1*, and *PmCHS1/2* (Figs 5A and Fig. S8B and C).

Additionally, we examined the methylation levels of key transcription factors (TFs) involved in the regulation of anthocyanin biosynthesis. The CG methylation levels in the gene bodies of alleles, such as *PmHY5* and *PmMYB308*, were significantly higher in the HM subgenome compared to the HC subgenome (Fig. S8A). In the promoter regions, the CG methylation levels of alleles including *PmMYB10.5*, *PmMYBR2*, *PmbHLH3*, *PmHY5*, *PmMYBR1*, and *PmMYB308* were also notably elevated in the HM subgenome relative to the HC subgenome. For the CHH context, methylation levels in the gene bodies of these transcription factors were generally low, whereas promoter regions exhibited significantly higher methylation, with detectable methylation occurring predominantly in the later developmental stages (Figs 5B and S8C).

During the leaf development of ‘Meiren’, the increasing levels of CHH methylation may be closely related to its growing environment. This study conducted correlation analyses between the methylation levels and expression of ABGs and transcription factors involved in anthocyanin biosynthesis. For most genes, CHH methylation levels gradually increased, peaking at the LS4 and LS5 stages, while gene expression levels progressively decreased, reaching their lowest points at LS4 or LS5, showing a negative correlation. Compared to the LS2 stage, CHH methylation levels in the coding or promoter regions of nearly all genes increased at LS4, but only 8 and 13 genes showed significant increases, respectively. Among the genes with significantly downregulated expression, 12 exhibited a negative correlation between methylation and expression (Fig. 5C). When comparing LS5 to LS1, CHH methylation levels increased in the coding regions of all ABGs and key transcription factors, with 20 genes showing significant differences. Similarly, CHH methylation levels rose in the promoter regions of all ABGs, with 37 genes displaying significant changes. Among the genes with significant methylation differences in either coding or promoter regions, 17 were downregulated. Five genes exhibited methylation differences in both coding and promoter regions alongside downregulated expression (Fig. 5D). In summary, the findings suggest that the transcriptional abundance of certain genes may be influenced by high methylation levels in their coding or promoter regions. In particular, increased methylation in promoter regions during prolonged exposure to rising temperatures and light intensity may play a role in regulating the transition from purple to green leaves in ‘Meiren’.

Differential CG methylation in both gene bodies and promoter regions may contribute to allelic expression bias, potentially affecting the regulation of anthocyanin biosynthesis during leaf development. Further correlation analysis between methylation levels and gene expression revealed that the *PmCHI1* alleles exhibited consistent ASM patterns in CG-type methylation, which showed an inverse relationship with expression trends (Fig. 5E). In ‘Meiren’, our previous study identified *PmMYB10.5b* as the central regulatory gene controlling anthocyanin biosynthesis [31]. The two allelic genes, derived from distinct subgenomes, exhibited consistent ASE patterns, with *PmMYB10.5b* demonstrating particularly high expression in leaves and fruits [31]. Notably, we observed significant epigenetic differences between these alleles. In the promoter region, *PmMYB10.5a* showed substantially higher methylation levels across all contexts (CG, CHG, and CHH) compared to *PmMYB10.5b*. Most strikingly, CG methylation in *PmMYB10.5a* displayed clear ASM patterns during both LS2 and LS5 developmental stages, and this methylation showed a negative correlation with gene expression levels (Fig. 5F). This methylation divergence was especially pronounced in the 1000-bp promoter region, where marked differences in methylation site density were observed (Fig. S9). The stage-specific ASM patterns in *PmMYB10.5* alleles suggest a developmentally regulated epigenetic mechanism that could affect pigment accumulation in ‘Meiren’ leaves. In contrast to the promoter region, the gene body exhibited no significant methylation differences between alleles, although both alleles showed a progressive increase in CHH methylation throughout development (Fig. 5F). So, we proposed that the observed inter-subgenome differences in CG methylation, particularly within the promoter region, likely play a crucial regulatory role in generating the characteristic purple leaf coloration in ‘Meiren’. The inverse relationship between promoter CG methylation and *PmMYB10.5b* expression may function as a key transcriptional regulatory mechanism that controls anthocyanin production and consequently, leaf pigmentation.

### Two alternative splicing variations were identified in the *PmMYB10.5b*

During the cloning of the *PmMYB10.5* gene coding sequence, we also identified multiple sequence mutation types, leading to variations in amino acid translation. The first type involved insertion mutations, where a 24-bp insertion resulted in an extended amino acid sequence of 252 aa. The second mutation type consisted of 10-bp deletions, which disrupted translation, yielding a truncated protein of 108 aa. Genomic sequence alignment revealed that the insertion–deletion (InDel) mutations occurred at the same site: the first exon's 124th position (CAAAGCAG[InDel][G/A]TGCA). The inserted fragments originated from the first intron sequence, while the deleted segments corresponded to the first 10 bp of the second exon (Fig. S10). Based on these findings, the 24-bp insertion variant of *PmMYB10.5b* was designated as *PmMYB10.5b1* (*PmMYB10.5b^△I24^*), and the 10-bp deletion variant was named *PmMYB10.5bP* (*PmMYB10.5b^△D10^*).

**Figure 6:**
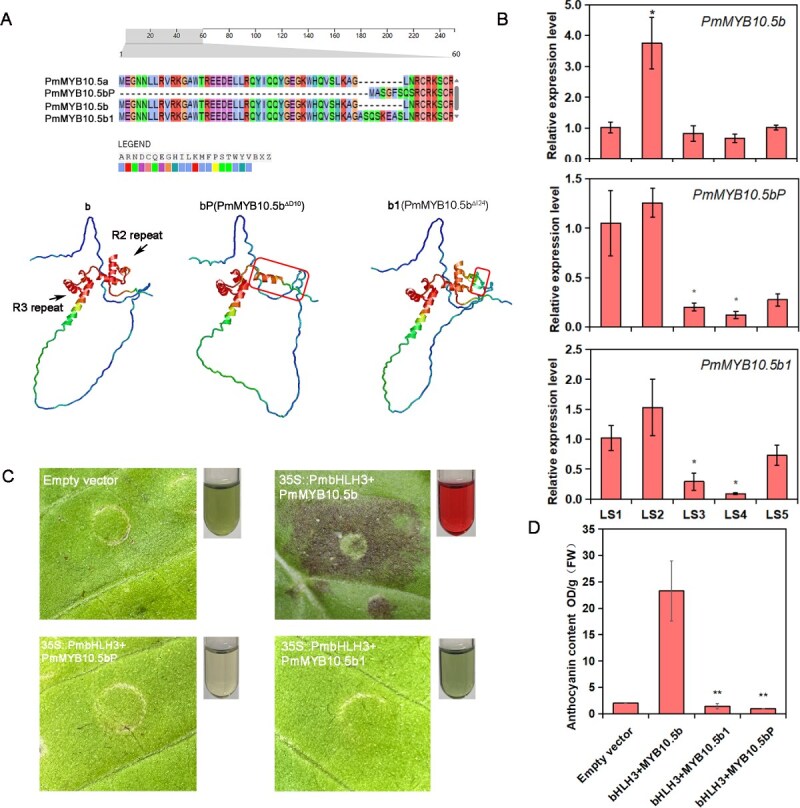
Functional validation of PmMYB10.5bP and PmMYB10.5b1. (A) Amino acid mutation sequences (PmMYB10.5bP, PmMYB10.5b1) of PmMYB10.5b (b) in R2 repeat domain. The 3D structures of PmMYB10.5b(b) and mutated proteins PmMYB10.5bP (bP) and PmMYB10.5b1 (b1) predicted by AlphaFold2 and viewed with RalMol v2.7.3 software. (B) The expression of *PmMYB10.5bP* and *PmMYB10.5b1*at the five developmental stages. (C) Transient overexpression phenotypes of *PmMYB10.5b* and mutant genes with *PmbHLH3* in tobacco leaves. (D) Anthocyanin content of transient overexpressed tobacco leaves in (C). Data are presented as mean ± s.d. of three biological replicates. One-tailed *t* test was used for statistical significance test (^*^P < 0.05, ^**^P < 0.01).

Then, we aligned the amino acid sequences of PmMYB10.5b, PmMYB10.5b1, and PmMYB10.5bP and predicted their three-dimensional structures using AlphaFold2. The results revealed that the InDel mutations directly altered the length and helical structure of the R2 motif. Specifically, PmMYB10.5bP nearly lost its entire R2 motif. PmMYB10.5b1 gained an 8-amino acid insertion (-ASQSKEAS-) between G [42] and L [43] sites, which likely affects its regulatory function (Fig. 6A). The phylogenetic analysis revealed that both PmMYB10.5bP and PmMYB10.5b1 clustered together with PmMYB10.5b, belonging to the S6 subgroup of the MYB transcription factor family, which is associated with the anthocyanin biosynthesis pathway (Fig. S11A). Subcellular localization analysis demonstrated that these mutant proteins were exclusively localized in the nucleus (Fig. S11B), consistent with the observed nuclear localization pattern of PmMYB10.5b [31]. We designed the specific qRT-PCR primers based on mutation sites and detected the expression levels of *PmMYB10.5bP* and *PmMYB10.5b1* at five stages of leaf development. The results showed that *PmMYB10.5bP* and *PmMYB10.5b1* had similar expression patterns to *PmMYB10.5b* [31], with an increase until the LS2 stage and a gradual decrease in expression as the leaves turning green (Fig. 6B). Moreover, their expression levels were significantly lower in the LS3 and LS4 stages compared to LS1, indicating that their functions may be related to the leaf color change.

### Both of two mutants fail to promote anthocyanin biosynthesis, instead act functions as an inhibitor

**Figure 7:**
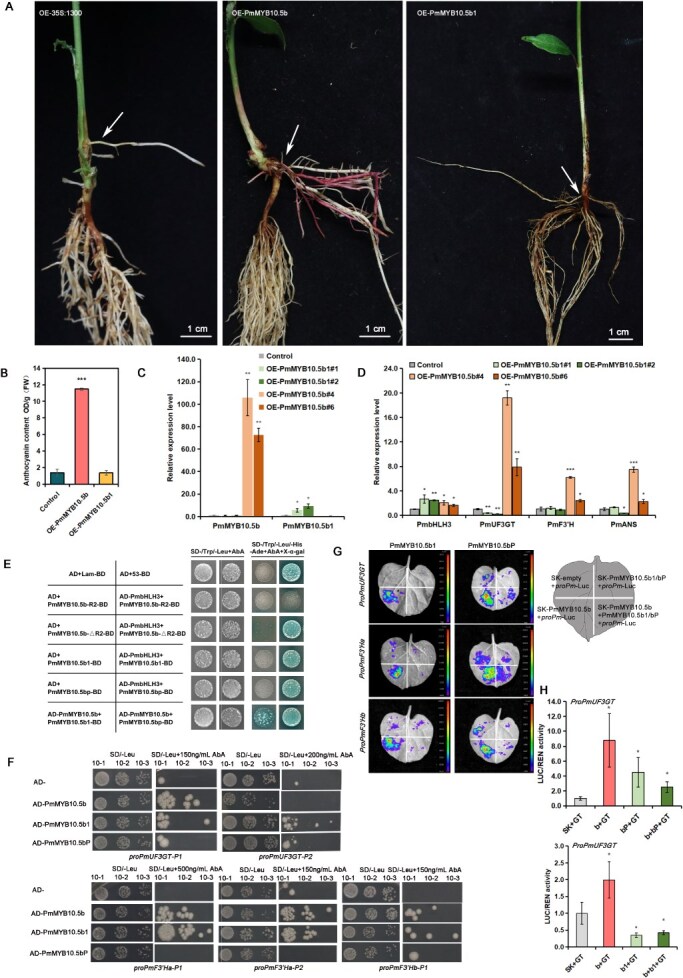
Molecular regulatory mechanism of PmMYB10.5bP and PmMYB10.5b1. (A) Hairy roots grow in the node round cotyledon overexpressing PmMYB10.5b1, PmMYB10.5b1, and empty vector. OE-PmMYB10.5b lines deposited pigment accumulation, OE-PmMYB10.5b1 lines did not deposit pigment accumulation. The white arrows represent the transgenic hairy roots. (B) Anthocyanin contents of the hairy roots of OE-PmMYB10.5b1, OE-PmMYB10.5b and control (empty vector) lines in *P. mume* seedings. (C) The expression of *PmMYB10.5b1* and *PmMYB10.5b* in OE and control lines. (D) The expression of *ABGs* and *PmbHLH3* in OE and control lines. (E) Y2H assay results of PmMYB10.5b mutant proteins. The concentration of AbA used in this study was 200 ng/mL. Positive control, AD +53-BD; Negative control, AD + Lam-BD. R2-BD presents the sequences of PmMYB10.5b R2 domain in 1–61aa, △R2-BD presents the sequences of PmMYB10.5b in 62–243aa without R2 domain. (F) Y1H assay results of PmMYB10.5b mutant proteins binding to the motifs of *proPmUF3GT*/*proPmF3′Ha*/*proPmF3′Hb*. (G) Activation of dual-luciferase reporter genes by two mutant proteins targeting the promoters of ABGs alleles. PmMYB10.5bP and PmMYB10.5b1 reduced the affinities of PmMYB10.5b binding to *proPmUF3GT*/*proPmF3′Ha*/*proPmF3′Hb*. A color bar is included to represent the range of luciferase intensity, with blue indicating weak expression and red indicating strong expression. (H) Dual-luciferase values of *PmMYB10.5b1*(b1) *and PmMYB10.5bP* (bP) binding to *proPmUF3GT* (GT). SK+ *proPmUF3GT* as the negative control, SK-PmMYB10.5b (b) + *proPmUF3GT* as the positive control. Data of B, C, D, H present mean ± SD of three biological replicates. One-tailed t-test was used for statistical significance test (^*^P < 0.05, ^**^P < 0.01, ^***^P < 0.0001).

Furthermore, we investigated whether the two identified InDel mutations affect their ability to activate anthocyanin biosynthetic enzyme gene expression and promote anthocyanin synthesis. Firstly, we performed transient overexpression of two mutants' co-infiltration with *PmbHLH3* in tobacco leaves. The results demonstrated that neither *PmMYB10.5b1* nor *PmMYB10.5bP* could induce anthocyanin accumulation when co-infiltrated with *PmbHLH3*, showing phenotypes difference to the *PmMYB10.5b* (Fig. 6C and D). This indicates that InDel mutations occurred in the R2 domain abolished the transcriptional activation function of PmMYB10.5b. Besides, we also investigated the regulatory role of *PmMYB10.5b1* in *P. mume* with hairy root transformation system we successfully constructed. Compared with the overexpressed *PmMYB10.5b* (OE-*PmMYB10.5b*), the OE-*PmMYB10.5b1* lines failed to accumulate extensive red pigments in hairy roots (Fig. 7A and B). The transcription levels of *PmMYB10.5b1* were significantly increased in OE-*PmMYB10.5b1* lines rather than in the OE-*PmMYB10.5b* and control lines (Fig. 7C). Overexpression of *PmMYB10.5b1* did not lead to the upregulation of the expressions of ABGs, such as *PmUF3GT*, *PmF3′H*, *PmANS* (Fig. 7D). These results suggested that R2 motif mutation of *PmMYB10.5b* caused by alternative splicing, making it unable to promote anthocyanin biosynthesis.

To further investigate how they participate in regulating the synthesis of anthocyanins, we firstly conducted Y2H assays to analyze whether their interaction functions were altered. The full-length protein sequence of *PmMYB10.5b,* two segments truncated with R2 domain or not (R2-BD, △R2-BD) were analyzed the interaction with PmbHLH3. The R2 domain did not interact with PmbHLH3, while △R2-BD could interact with PmbHLH3, consistent with the existing researches that interaction site of MYBs and bHLHs is located in R3 domain, not R2 [33, 34]. The results confirmed that both mutants maintained their ability to interact with PmbHLH3, suggesting that the R2 motif mutations do not affect the interaction of MBW complex (Fig. 7E). Then, we hypothesize that the loss-of-function in R2 mutants may result from either impaired DNA-binding ability to ABG promoters or conversion into transcriptional repressors. Y1H assays indicated that the two mutants can still bind to the ABGs (*PmUF3GT*, *PmF3′H* alleles) promoters, especially, the binding ability of PmMYB10.5b1 was almost the same PmMYB10.5b (Fig. 7F). Dual-luciferase reporter assays supported that PmMYB10.5b could bind to the ABGs promoters to activate its expression. While, both PmMYB10.5b1 and PmMYB10.5bP exhibited transcriptional repression activity when co-expressed with PmMYB10.5b (Fig. 7G and H). In summary, alternative splicing mutations in the *PmMYB10.5b* lead to a loss of their anthocyanin activation function and may act as competitive inhibitors or potential repressors, which can still interact with the PmbHLH3 and bind to the ABGs promoter, thereby inhibiting their expression and anthocyanin biosynthesis. The exact molecular mechanism underlying this effect requires further elucidation.

## Discussion

Both epigenetic modifications and mRNA alternative splicing are closely linked to environmental factors. Plants respond to environmental changes and stimuli through mechanisms such as epigenetic regulation and post-transcriptional control [26, 35]. DNA methylation, an epigenetic modification, can regulate gene expression at the transcriptional level. In horticultural crops like tea plants, methylation plays a role in growth and development under environmental variations, affecting fruit quality, ripening, coloration, secondary metabolites, and stress resistance [36–40]. In ‘Meiren’, leaf color differences during development are influenced by seasonal environmental changes. Observations reveal that leaves are deep purple during early spring bud break. As shoots elongate and leaves expand, the lower leaves gradually turn green. Under the high temperature and intense light of summer, the upper leaf surfaces begin greening until the leaves completely revert to green, showing distinct seasonal variation. To investigate the impact of methylation on leaf coloration, we performed whole-genome methylation sequencing on leaves at different color stages. The study found that global DNA methylation levels in ‘Meiren’ gradually increased as the purple leaves gradually fade to green with increasing summer temperatures, particularly with differential at CHH methylation in promoter and gene region of anthocyanin biosynthesis genes. In contrast, CG methylation shows no significant fluctuations during leaf development but exhibits substantial differences between the HM and HC subgenomes, indicating subgenomic imbalance. This methylation imbalance (ASM) can also lead to expression differences in alleles (ASE), thereby affecting phenotype [41].

Our analysis further revealed a negative correlation between expression levels and methylation status for certain DMGs, particularly evident in some ASEGs. Notably, we observed significant methylation variations in both gene bodies and promoter regions of anthocyanin biosynthesis genes, with the most pronounced differences occurring at CG sites. A striking allelic difference was detected in the *PmMYB10.5* gene, where the *PmMYB10.5a* allele showed significantly higher promoter methylation (especially at CG sites) compared to PmMYB10.5b, correlating with its reduced expression level. During late leaf development, all anthocyanin biosynthetic genes exhibited increased CHH methylation coupled with decreased expression levels. These findings strongly support the regulatory relationship between DNA methylation patterns (particularly CG and CHH contexts) and gene expression differences, including ASE. In Rosaceae plants, hypermethylation of promoter regions in S6-clade MYB transcription factors leads to loss in purple-red traits [42–44], suggesting that high methylation levels can suppress the expression of activators, thereby inhibiting anthocyanin biosynthesis. We propose that both the observed CG methylation imbalance between alleles and the dynamic CHH methylation changes during development may contribute to two key phenomena: the formation of heterosis in interspecific hybrids and the purple leaf phenotype variation in ‘Meiren’. However, the precise mechanistic links between these epigenetic modifications and phenotypic outcomes warrant further in-depth investigation to fully understand their roles in hybrid vigor and trait regulation.

On the other hand, alternative splicing has been shown to participate in plant immune responses and environmental adaptation [45]. However, existing research also indicates that alternative splicing can influence anthocyanin biosynthesis. In this study, we identified and successfully cloned two coding sequence InDel mutations (*PmMYB10.5b1* and *PmMYB10.5bP*) in the *PmMYB10.5b* gene from ‘Meiren’. Sequence analysis suggested these mutations likely represent distinct alternative splicing variants of mRNA. The mutation sites are located in the first and second introns, featuring a 24-bp insertion (*PmMYB10.5b1*) and a 10-bp deletion (*PmMYB10.5bP*), respectively, both of which alter the coding sequence and consequently lead to changes in protein translation and structure. Functional characterization revealed that both *PmMYB10.5b1* and *PmMYB10.5bP* lose their transcriptional activation capability, failing to promote anthocyanin synthesis. Dual-luciferase reporter assays further indicated that these mutations may confer repressive activity, as both the 24-bp insertion and 10-bp deletion disrupt critical amino acid residues downstream of position 42 in the R2 motif of PmMYB10.5b. This suggests that this region serves as a key determinant for the functional switch between activator and repressor roles in PmMYB10.5b. Notably, similar mutations have been reported in the *FaMYB5* gene of strawberry [46]. In grapes, *VvMYBA1* acts as a transcriptional activator promoting anthocyanin accumulation in flesh, whereas its alternatively spliced variant VvMYBA1-L inhibits coloration consistent with the findings for *PmMYB10.5b* in ‘Meiren’, though the splicing sites differ [47]. In cornflower (*Centaurea cyanus*), alternative splicing of *CcbHLH1* leads to loss of activation function, resulting in complete absence of anthocyanins in white petals [48]. This function and regulatory mechanism are similar to the R2R3-MYB repressors located at S4 clade [49], which may interact with PmbHLH3, and compete with PmMYB10.5b for PmbHLH3 interaction to inhibit the expression of ABGs. However, further research will be required to determine the specific inhibitory mechanisms of the two mutants. In addition, we investigated the mechanism of leaf color response to environmental factors by treating ‘Meiren’ leaves with high-temperature and light, and will further explore the function of alternative splicing of *PmMYB10.5b* under this condition.

## Conclusion

In summary, we performed WGBS on ‘Meiren’ leaves across developmental stages that displayed a range of purple color. A significant increase in CHH methylation levels was observed during leaf development. Notably, we uncovered ASM in CG methylation between its two haplotypes, with the hypermethylated HM subgenome showing downregulated expression of key anthocyanin genes, including *PmMYB10.5*. Furthermore, we functionally characterized two alternative splicing variants of *PmMYB10.5b* (*PmMYB10.5b1* and *PmMYB10.5bP*), which arise from InDel mutations disrupting the R2 domain. While the two mutations retained their ability to interact with PmbHLH3 and bind to the ABGs promoter, they lost the function of promoting anthocyanin biosynthesis. These variants may act as repressors by competing with the full-length MYB protein for bHLH interaction, thereby inhibiting the anthocyanin biosynthesis. This study provides new genetic and epigenetic perspectives on purple leaf change in woody plants.

## Materials and methods

### Plants materials

A mature *Prunus mume* ‘Meiren’ tree cultivated at Beijing Jiu Feng National Forest Park (Beijing, China) served as the plant material. We selected five different developmental stages of leaves exhibiting distinct color differences described in our previous study: undeveloped young apical leaves (LS1), apical leaves (LS2), upper middle leaves (LS3), lower middle leaves (LS4), and mature apical leaves (LS5) for RNA-seq and bisulfite-seq analysis [31]. Genomic DNA was isolated using the DNAprep Pure Plant Kit (TIANGEN, Beijing, China). All samples of ‘Meiren’ leaves were collected from the same tree, with three biological replicates per stage.

### RNA-seq analysis and ASE identification

RNA-seq and ASE analysis were conducted as previously reported [31]. Briefly, the haplotype-resolved genome of ‘Meiren’ was used as a reference to identify allelic gene pairs. Transcriptomes from leaves at the five developmental stages were sequenced, and ASEGs were characterized.

### Bisulfite-seq library preparation and sequencing

Whole-genome bisulfite sequencing (WGBS) was also performed on ‘Meiren’ leaf samples. Genomic DNA (5 μg) was spiked with 25 ng lambda-DNA, fragmented to ~300 bp using a sonicator (Sonics & Materials), and subjected to bisulfite conversion with the ZYMO EZ DNA Methylation-Gold kit (ZYMO). Then, the qualified bisulfite-converted libraries were sequenced on an Illumina HiSeq2500 platform, generating approximately 6 Gb of 150 bp paired-end reads per sample (Shanghai BIOZERON Co., Ltd).

### WGBS data analysis

Raw WGBS reads were quality-assessed using FastQC v0.11.5 [50] and trimmed using the Trimmomatic v0.36 [51] with parameters (SLIDINGWINDOW:4:15 MINLEN:75). Processed reads were mapped to the ‘Meiren’ haplotype-resolved genome with BSMAP aligner allowing up to two mismatches [52]. Uniquely mapped reads were used to determine the cytosine methylation levels, and individual cytosines with more than four reads were considered for DNA methylation-level calculation. Then, a Perl script was employed to compute methylation levels.

### The DMRs identification

Differential methylation cytosines (DMCs) and DMRs were identified as previously described [53]. Only cytosines covered by at least four reads in a library were included. DMRs were detected using a 200-bp sliding-window moving in the 50-bp step-size. Pairwise comparisons of methylations at different leaf developmental stages were conducted using Fisher's exact test, with *P*-values adjusted via the Benjamini–Hochberg method. Windows with an FDR < 0.05 and a methylation fold change (FC) ≥ 1.5 were selected for further analysis. Moreover, individual cytosines within these windows were considered DMCs if they met the following thresholds: *P* ≤ 0.01, FC ≥ 2, and absolute methylation difference ≥ 0.4 (CG), ≥ 0.2 (CHG), or ≥ 0.1 (CHH). DMRs were retained only if they contained at least 7 DMCs and, adjacent DMRs separated by ≤100 bp were merged.

Functional annotation of genes overlapping with DMRs was performed through GO term and KEGG pathway enrichment analyses using Goatools [54] (https://github.com/tanghaibao/Goatools) and KOBAS [55] (http://kobas.cbi.pku.edu.cn/kobas3). Terms with a Bonferroni-corrected *P*-value <0.05 were deemed significantly enriched.

### Vectors construction and transient overexpression in tobacco

The full-length coding sequences of InDels named *PmMYB10.5b1* and *PmMYB10.5bP* were amplified from the ‘Meiren’ leaf cDNA using the PrimeSTAR ® HS DNA Polymerase (TaKaRa, Japan). The multiple sequence alignment was performed by the MAFFT [56] and DNAMAN v9. Phylogenetic analysis was conducted by aligning protein sequences with ClustalW and constructing neighbor-joining trees in MEGA7 (1000 bootstrap replicates, *P*-distance method) [57]. Protein 3D structures were predicted using AlphaFold2 [58] and viewed in RasMol v2.7.3 software.

For genetic transformation and subcellular localization, target genes were subcloned into the *XbaI*- and *SpeI-* sites of the pSuper1300-GFP vector, generating *CaMV35S*-driven pSuper1300-gene-GFP constructs. These were transferred into *Agrobacterium tumefaciens* strain GV3101(WeiDi, Shanghai, China) for Agrobacterium-mediated transformation. Transient transformation for tobacco (*N. tabacum*) leaves was carried out according to the previous research [49]. Briefly, following PCR verification of the positive colonies, the bacteria were cultured overnight in LB medium supplemented with 50 mg/L Kanamycin (Kan) and 50 mg/L Rifampicin (Rif) until the OD_600_ reached about 0.8. The bacterial suspensions were then resuspended in an equal volume of infiltration buffer (10 mM MgCl_2_, 150 μM acetosyringone [AS]), and 10 mM MES ((2-(N-morpholino) ethanesulfonic acid), pH 5.6), incubated at room temperature in darkness for 3 h, and then injected into the abaxial side of tobacco leaves. After 12 to 24 h of dark incubation, plants were moved to a 16/8-h light/dark cycle at 22°C. Phenotypes were documented after 5 days.

### Dual luciferase assay

The pGreenII 62-SK and pGreenII 0800-Luc systems were used for dual-luciferase assays. The target proteins were subcloned into the SK vector, and promoters were subcloned into the LUC reporter system to construct recombinant plasmids. Recombinant plasmids were separately transformed into *A. tumefaciens* GV3101 strains harboring the pSoup helper plasmid (Weidi, Shanghai, China) and incubated at 28°C for 3 days. The transformed positive cultures were resuspended in infiltration buffer (10 mM MgCl₂, 200 μM AS, and 10 mM MES; OD_600_ = 0.8) and incubated at 25°C for 3 h before infiltration into young leaves of 4-week-old *N. benthamiana* seedlings. As a control, the *proPm*-LUC reporter was co-infiltrated with the SK empty vector. After 24 h in darkness, the plants were returned to normal growth conditions. Luciferase activity was assayed 48 h post-infiltration by applying a luciferin solution (0.1% Triton X-100, 0.32 mg/ml d-luciferin potassium salt) to the abaxial leaf surface. The LUC fluorescence intensity was visualized and photographed by a BERTHOLD NightOwl II LB 983 imaging system (Berthold Technologies, Germany). LUC activity value detection was referenced as our previous study [31].

### Y2H and Y1H

To validate protein–protein interactions, we employed the Y2H using pGADT7 (AD) and pGBKT7 (BD) vectors [31]. Target genes were individually subcloned into AD and BD vectors to construct recombinant plasmids. The BD constructs were transformed into Y187 yeast cells, and the AD constructs were transformed into Y2H Gold cells, following the manufacturer's protocol (WeiDi, Shanghai; Cat# YC1020 and YC1002). Transformants were selected on SD/-Trp medium and grown at 30°C for 2 to 4 days. To test for autoactivation, BD-transformants were serially diluted (10×, 100×, and 1000×) and spotted on SD/-Trp plates containing 0–200 ng/mL Aureobasidin A (AbA). For interaction tests, 15 μL each of AD-target (Y2H Gold) and BD-target (Y187) yeast suspensions were mixed with 90 μL YPDA in a culture plate and incubated at 30°C with 200 rpm shaking for 24 h. The mated cultures were then diluted (10× and 100×), and 5 μL aliquots were spotted onto SD/-Trp/-Leu and SD/-Trp/-Leu/-His/-AbA+X-α-Gal plates. After 2 to 4 days at 30°C, yeast growth and blue color development were assessed to evaluate interactions. The pGADT7-T + pGBKT7-Lam and pGADT7-T + pGBKT7-53 served as the negative and the positive control, respectively.

For Y1H, the promoter sequences of the target gene were cloned into the pAbAi vector to generate the pAbAi-pro vector. The plasmid was linearized with BstBI (NEB, Beijing) and integrated into the genome of Y1HGold yeast. Positive clones were selected on SD/-Ura plates and cultured at 30°C for 3 to 4 days. Subsequently, the minimum inhibitory concentration of aureobasidin A (AbA) for the reporter strain was determined to suppress HIS tag leakage. For the interaction assay, the coding sequence of the transcription factor was cloned into the pGADT7 vector. The resulting plasmid was transformed into the prepared Y1H strain with promoters using the Kit (YCK1010M, WeiDi, Shanghai, China). The transformants were selected on SD/-Leu plates and incubated at 30°C for 3 to 4 days. A single positive colony was inoculated in liquid SD/-Leu medium and grown to an OD_600_ of 0.2. The culture was then serially diluted (10×, 100×, and 1000×), and 5 μl of each dilution was spotted onto SD/-Leu plates containing the predetermined inhibitory concentration of AbA. The plates were incubated at 30°C for 3 to 4 days, and recorded to evaluate interactions. Primers involved in this study were shown in Table S4.

### Anthocyanin content determination

The total anthocyanin content was determined using a UV-spectrophotometric method with minor modifications. In brief, approximately 0.1 g of sample (ground under liquid nitrogen) was extracted with 1.5 mL of acidified methanol (1% HCl, v/v) in pre-cooled centrifuge tubes. The mixtures were kept at 4°C in darkness for 24 h until the tissues became colorless. Following centrifugation at 12,000 rpm for 10 min at 4°C, the absorbance of the supernatant was measured at 530 nm (for anthocyanins) and 654 nm (for correction) using a 1-cm cuvette on a Biomate 3S spectrophotometer (Thermo, Germany). The anthocyanin concentration was calculated as (A_530_-0.25 × A_654_) g^−1^ FW (fresh weight) [31, 59].

## Supplementary Material

Web_Material_uhag039

## Data Availability

The data underlying this article are available in the article and in its online supplementary material.
