# Supplementary material for: Allele-specific methylation, and InDels of PmMYB10.5b induced by alternative splicing, participate in regulating the leaf color change in Prunus mume ‘Meiren’
Source: Hortic Res. 2026 Feb 18;13(5):uhag039. doi: 10.1093/hr/uhag039 (PMC13161575; doi:10.1093/hr/uhag039)
Supplement: Web_Material_uhag039 [file web_material_uhag039.zip › 2-Supplementary Figures.docx]

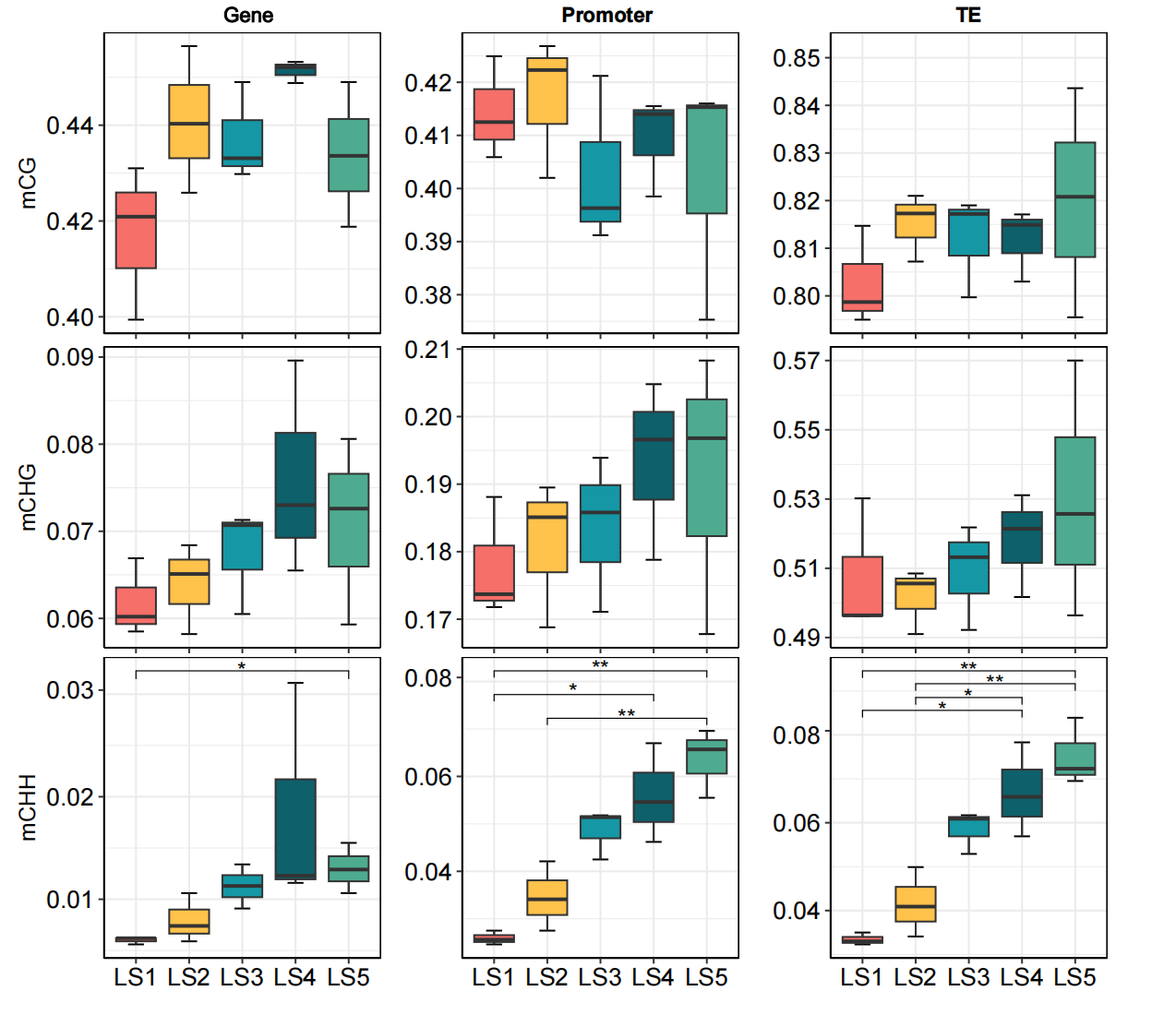


Fig.S1 Methylation levels of CG, CHG and CHH in gene bodies, TEs, and gene promoter regions.


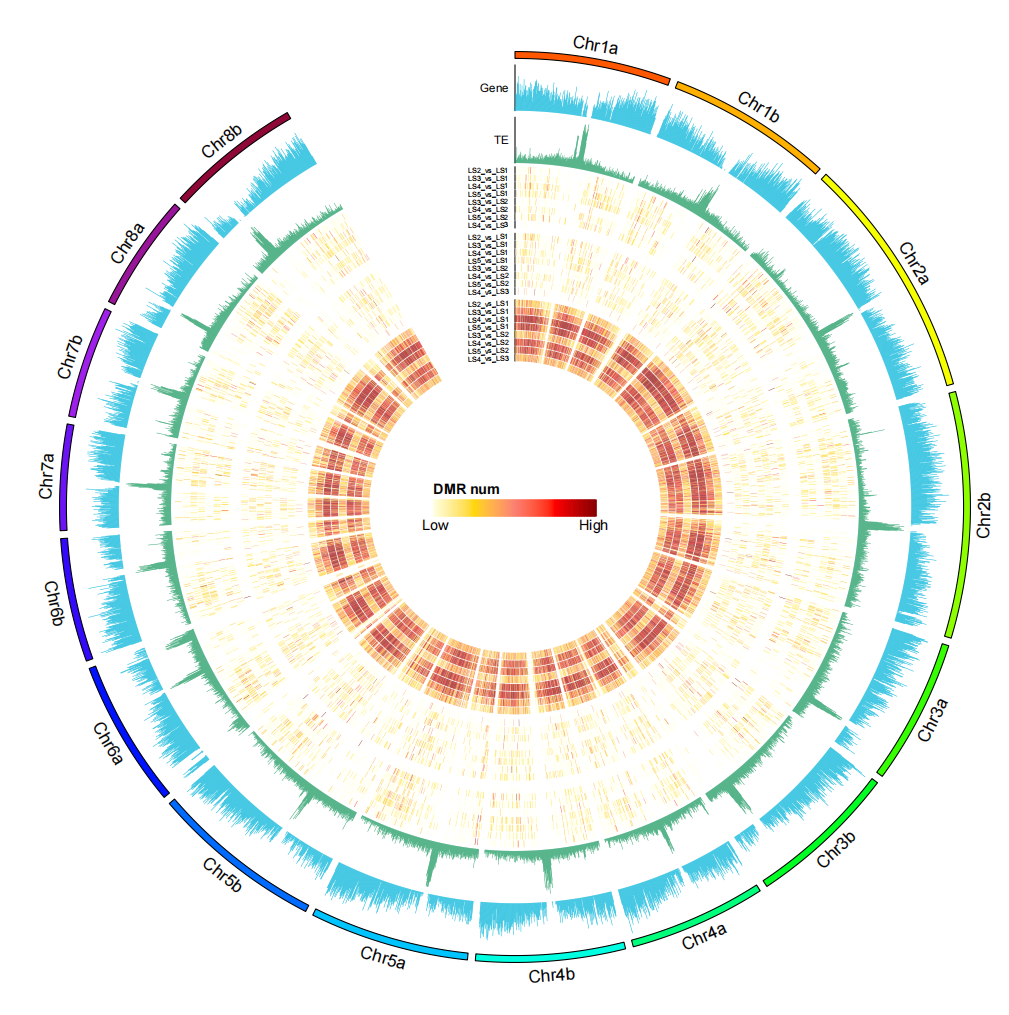


Fig.S2 Genome-wide distribution characteristics of DMRs in ‘Meiren’ leaf developmental stages.


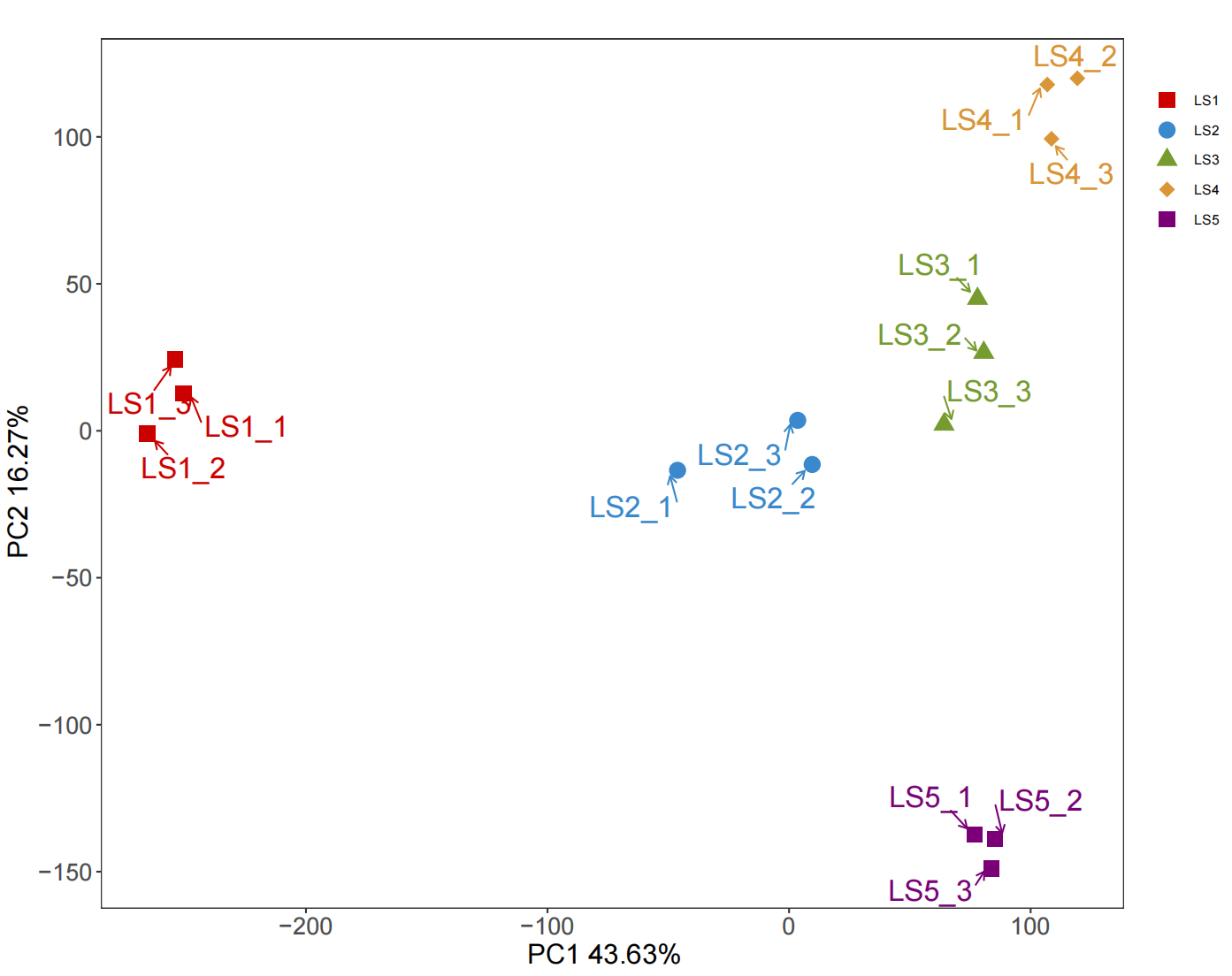


Fig.S3 PCA analysis of sample correlation between different developmental stages.


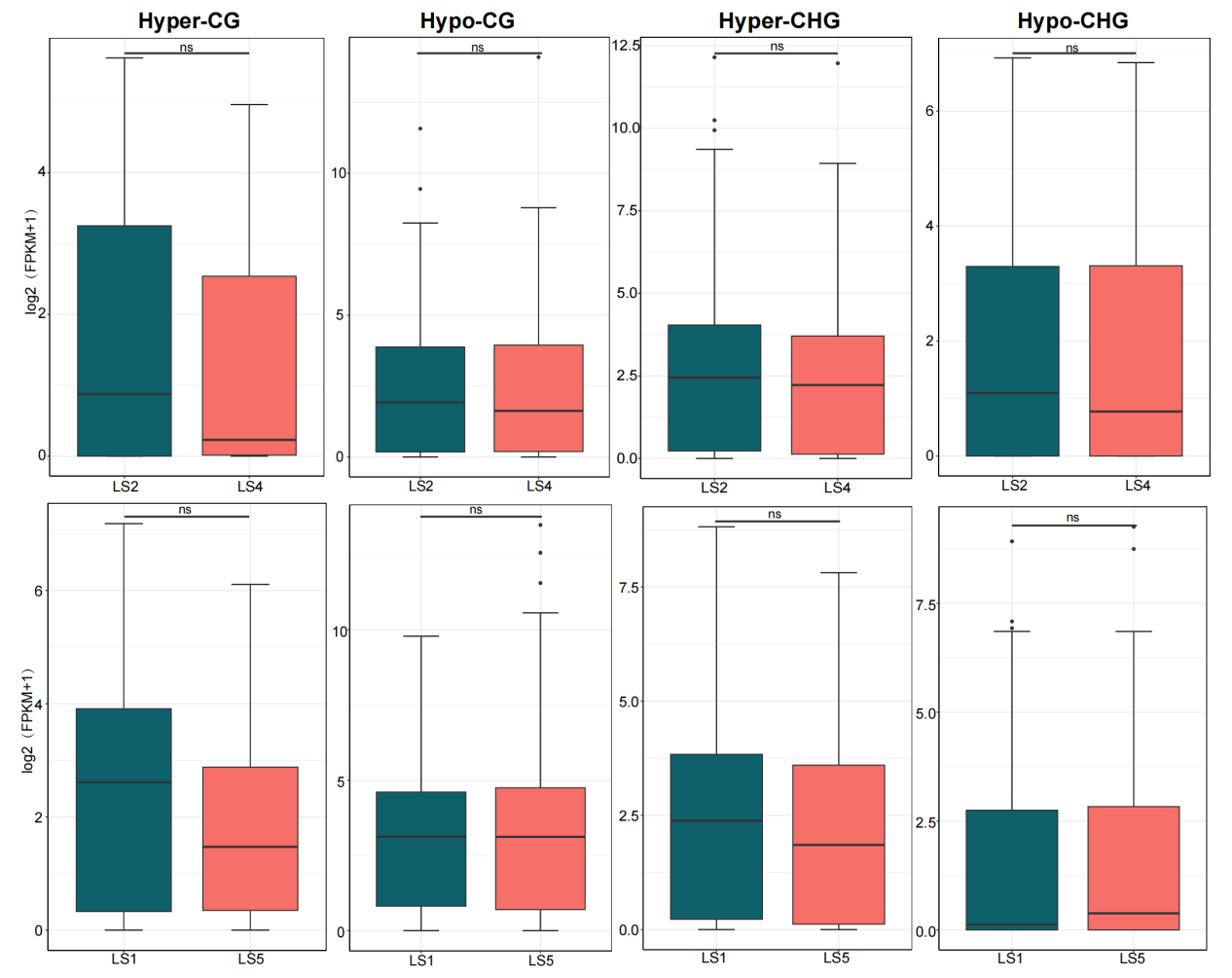


Fig.S4 The expression levels (log2(FPKM + 1)) of hyper- or hypo- CG and CHG DMGs at LS5 vs. LS1 and LS4 vs. LS2. The horizontal line within the box represents the median, box limits represent upper and lower quartiles, and whiskers represent 1.5 interquartile range.


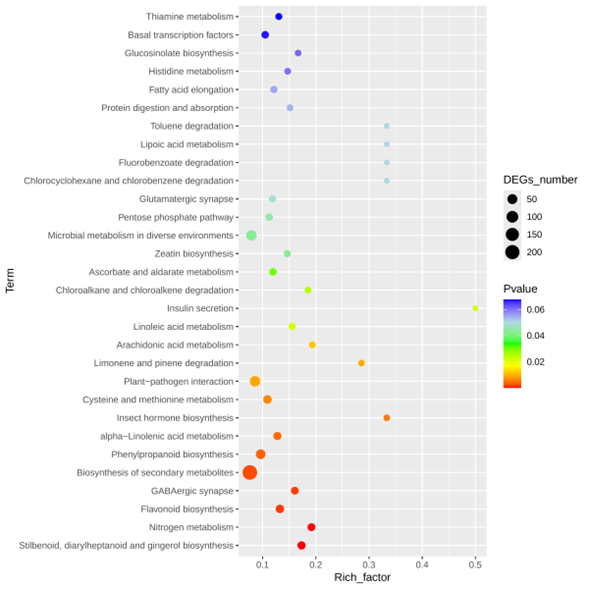


Fig.S5 KEGG functional enrichment of ASMGs in CG content at the LS5 stage.


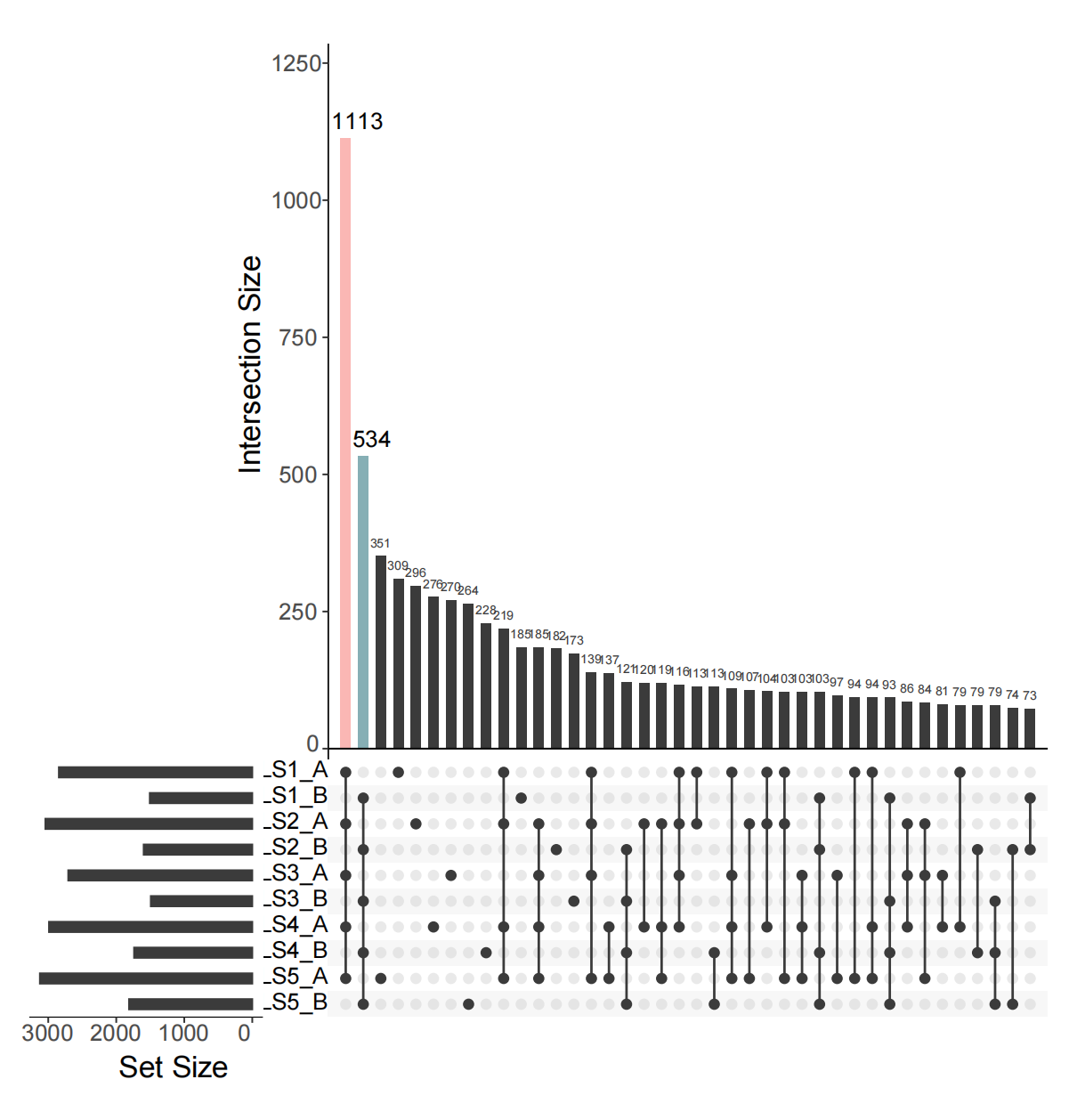


Fig.S6 CG allele-specific methylation (ASM) numbers of promoter 2k region(b) at 5 leaf developmental stages. The upset plot was drawn by UpSetR package. Only top 40 categories are shown. A, alleles were from HM. B, alleles were from HC.


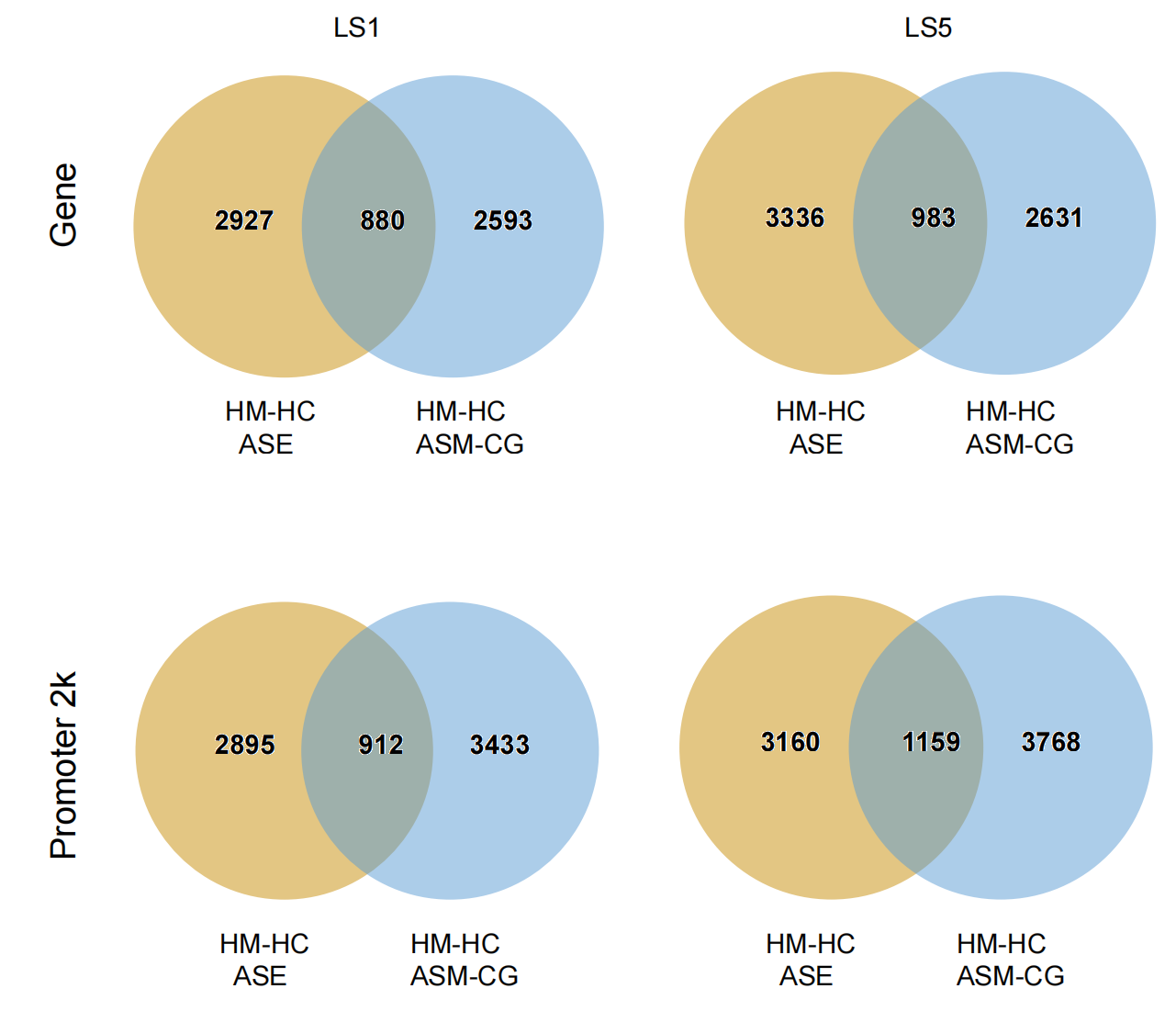


Fig.S7 Venn diagram showing the overlap between CG-specific methylation numbers and ASE numbers in gene regions and promoter regions of alleles.


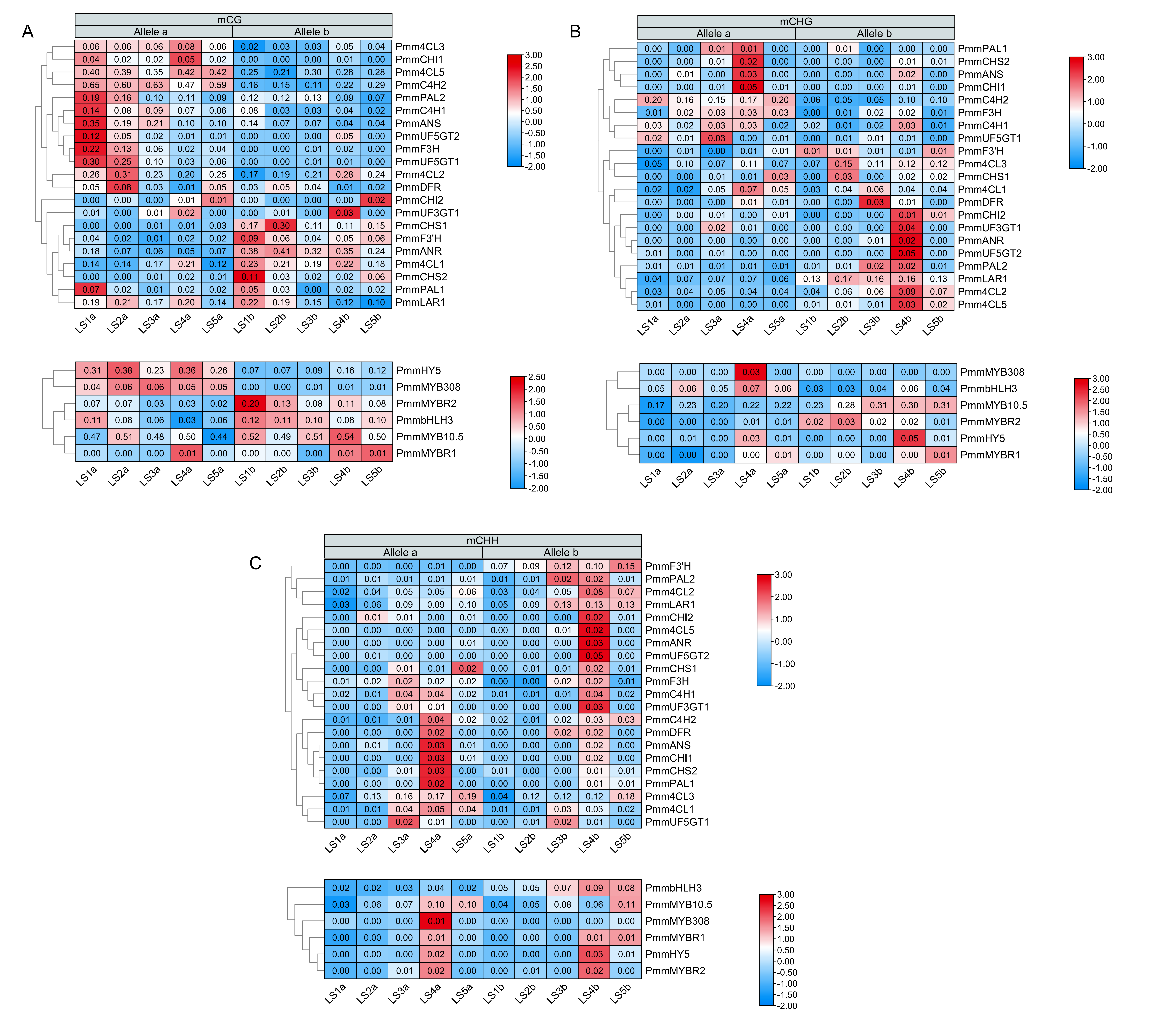


Fig.S8 Heatmap of mCG (A), mCHG (B) and mCHH (C) methylation levels in the gene body of ABGs and transcription factor alleles.


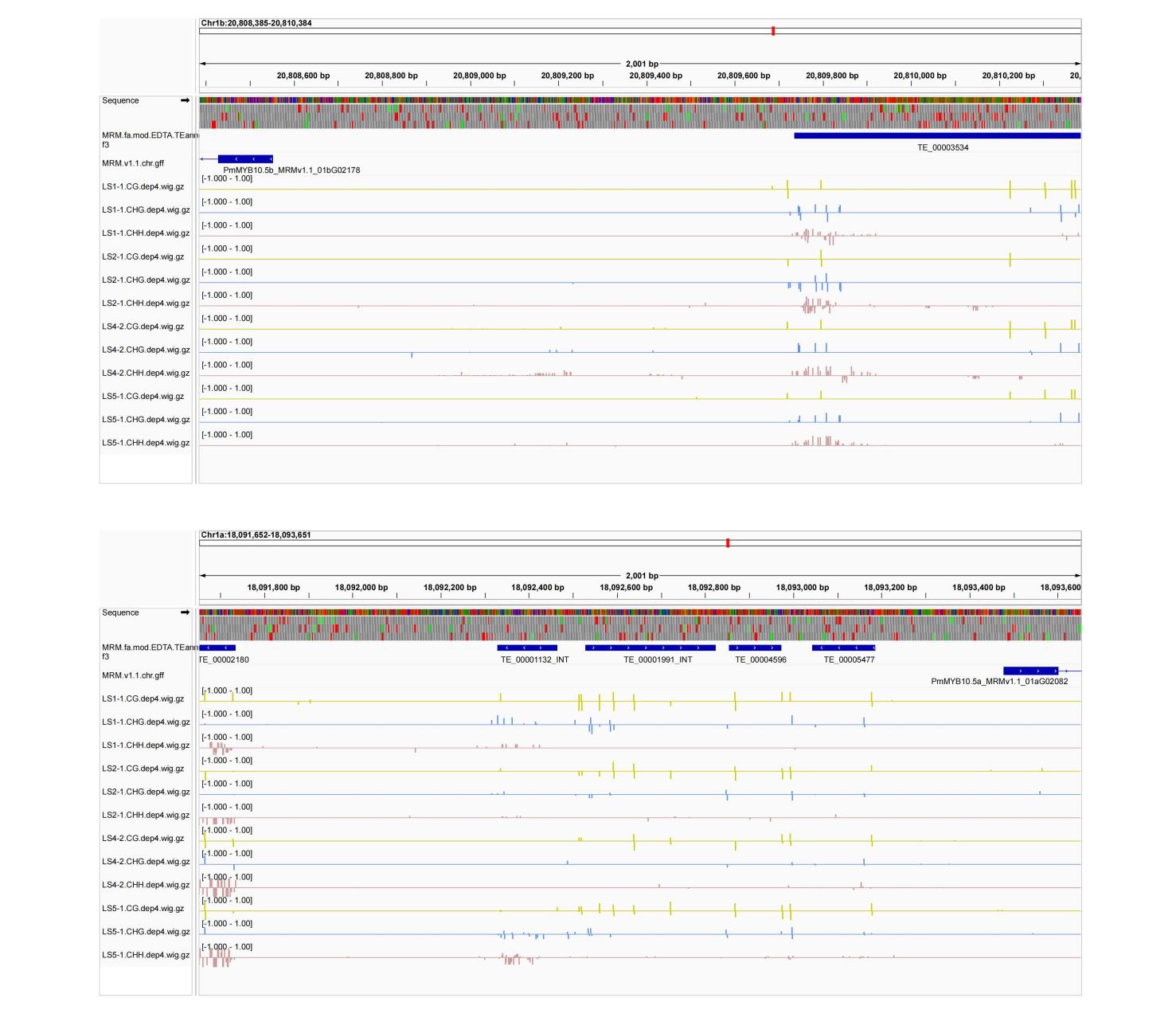


Fig.S9 CG methylation sites in the promoter regions of *PmMYB10.5* alleles.

Fig.S10 Multiple sequence alignment of coding sequences of *PmMYB10.5b* and its mutants.


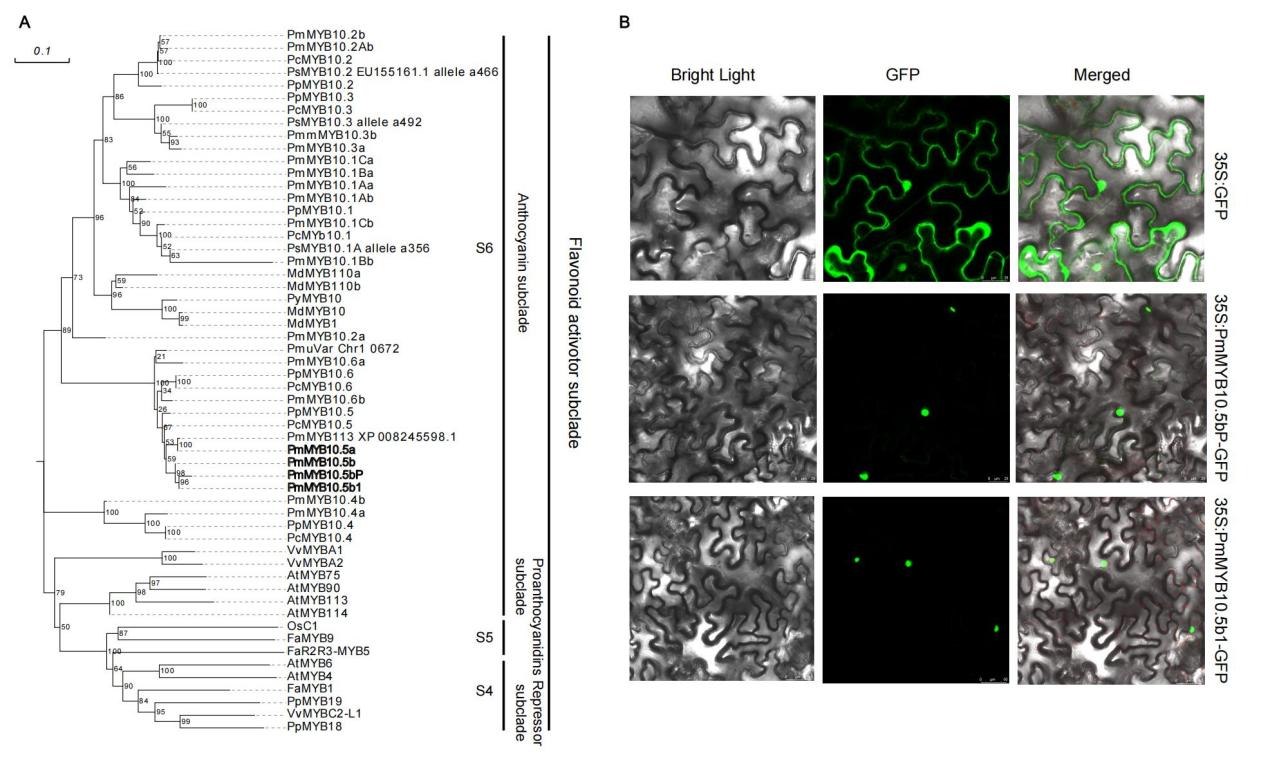
Fig.S11 Phylogenetic tree of PmMYB10.5 and mutated genes (A), as well as subcellular localization results of PmMYB10.5b1 and PmMYB10.5bP in tobacco leaves fused to GFP. Bars = 25μm in the figures (B). The amino acid sequences of other plants were retrieved from GenBank databases and Genome Database for Rosaceae (GDR, http://www.rosaceae.org/), including Arabidopsis (AtMYB75/PAP1 AT1G56650, AtMYB90/PAP2 At1g66390, AtMYB113 At1g66370, AtMYB114 At1g66380, AtMYB4 AT4G38620, AtMYB6 AT4G09460), *Oryza sativa* (OsC1 HQ379703), *Vitis vinifera* (VvMYBA1 AB097923, VvMYBA2 AB097924, VvMYBC2-L1 JX050227), *P. persica* (PpMYB10.1–10.6 ppa026640m, ppa016711m, ppa020385m, ppa018744m, ppa022808m, ppa024617m, PpMYB19 KT159235, PpMYB18 KT159234), *P. salicina* (PsMYB10.1A allele a356, PsMYB10.3 allele a492, PsMYB10.2 EU155161.1 allele a466), *P. cerasifera* (PcMYB10.1–10.6 KP772281, KP772282, KP772282, KP772284, KP772285, KP772286), *P. mume* (PmuVarChr10672, PmMYB113 XP 008245598.1), *Fragaria x ananassa* (FaMYB9 JQ989281, FaMYB1 AF401220, FaR2R3-MYB5 MW700311) and *Malus x domestica* (MdMYB110a JN711473, MdMYB110b JN711474, MdMYB10 DQ267897, MdMYB1 ABB84755).
